# A systematic umbrella review and meta-meta-analysis of eHealth and mHealth interventions for improving lifestyle behaviours

**DOI:** 10.1038/s41746-024-01172-y

**Published:** 2024-07-05

**Authors:** Ben Singh, Mavra Ahmed, Amanda E. Staiano, Claire Gough, Jasmine Petersen, Corneel Vandelanotte, Chelsea Kracht, Christopher Huong, Zenong Yin, Maria F. Vasiloglou, Chen-Chia Pan, Camille E. Short, Matthew Mclaughlin, Lauren von Klinggraeff, Christopher D. Pfledderer, Lisa J. Moran, Alyssa M. Button, Carol A. Maher

**Affiliations:** 1 Alliance for Research in Exercise Nutrition and Activity (ARENA), University of South Australia, Adelaide, SA Australia; 2 Department of Nutritional Sciences and Joannah and Brian Lawson Centre for Child Nutrition, Temerty Faculty of Medicine, University of Toronto, Toronto, ON Canada; 3 Pennington Biomedical Research Center, Louisiana State University, Baton Rouge, LA USA; 4 Flinders University, College of Nursing and Health Sciences, Adelaide, SA Australia; 5 Flinders University: College of Education, Psychology and Social Work, Adelaide, SA Australia; 6 Physical Activity Research Group, Appleton Institute, Central Queensland University, Rockhampton, QLD Australia; 7 Department of Public Health, University of Texas at San Antonio, San Antonio, TX USA; 8 Nestlé Institute of Health Sciences, Nestlé Research, 1000 Lausanne, Switzerland; 9 Leibniz Institute for Prevention Research and Epidemiology—BIPS, Bremen, Germany; 10 Department of Prevention and Health Promotion, Institute for Public Health and Nursing Research, University of Bremen, Bremen, Germany; 11 Melbourne Centre for Behaviour Change, Melbourne School of Psychological Sciences and Melbourne School of Health Sciences (jointly appointed), University of Melbourne, Parkville, VIC Australia; 12 Telethon Kids Institute, University of Western Australia, Perth, WA Australia; 13 Department of Community and Behavioral Health Sciences, Institute of Public and Preventive Health, School of Public Health, Augusta University, Augusta, GA USA; 14 Department of Health Promotion and Behavorial Sciences, University of Texas Health Science Center Houston, School of Public Health in Austin, Austin, TX USA; 15 Monash Centre for Health Research and Implementation, Monash University, Clayton, VIC Australia

**Keywords:** Public health, Weight management

## Abstract

The aim of this meta-meta-analysis was to systematically review randomised controlled trial (RCT) evidence examining the effectiveness of e- and m-Health interventions designed to improve physical activity, sedentary behaviour, healthy eating and sleep. Nine electronic databases were searched for eligible studies published from inception to 1 June 2023. Systematic reviews with meta-analyses of RCTs that evaluate e- and m-Health interventions designed to improve physical activity, sedentary behaviour, sleep and healthy eating in any adult population were included. Forty-seven meta-analyses were included, comprising of 507 RCTs and 206,873 participants. Interventions involved mobile apps, web-based and SMS interventions, with 14 focused on physical activity, 3 for diet, 4 for sleep and 26 evaluating multiple behaviours. Meta-meta-analyses showed that e- and m-Health interventions resulted in improvements in steps/day (mean difference, MD = 1329 [95% CI = 593.9, 2065.7] steps/day), moderate-to-vigorous physical activity (MD = 55.1 [95% CI = 13.8, 96.4] min/week), total physical activity (MD = 44.8 [95% CI = 21.6, 67.9] min/week), sedentary behaviour (MD = −426.3 [95% CI = −850.2, −2.3] min/week), fruit and vegetable consumption (MD = 0.57 [95% CI = 0.11, 1.02] servings/day), energy intake (MD = −102.9 kcals/day), saturated fat consumption (MD = −5.5 grams/day), and bodyweight (MD = −1.89 [95% CI = −2.42, −1.36] kg). Analyses based on standardised mean differences (SMD) showed improvements in sleep quality (SMD = 0.56, 95% CI = 0.40, 0.72) and insomnia severity (SMD = −0.90, 95% CI = −1.14, −0.65). Most subgroup analyses were not significant, suggesting that a variety of e- and m-Health interventions are effective across diverse age and health populations. These interventions offer scalable and accessible approaches to help individuals adopt and sustain healthier behaviours, with implications for broader public health and healthcare challenges.

## Introduction

In recent decades, the rise in chronic diseases has posed a complex challenge to global healthcare^1,2^. Conditions like obesity, cardiovascular diseases, type 2 diabetes, and mental health disorders are closely linked to behavioural practices^3^. Notably, 24-hr movement behaviours (physical inactivity, extended sedentary periods, insufficient sleep) and poor dietary choices are key modifiable risk factors for chronic diseases^4–8^. The global economic burden of chronic diseases is estimated at $47 trillion (USD) between 2010 and 2025^9^. Addressing these risk factors through healthier behaviours in physical activity, sedentary behaviour, sleep, and diet can significantly reduce chronic disease incidence and severity^3,10–13^.

The non-adherence rate to recommended health behaviours including physical activity and sedentary behaviour (e.g., World Health Organization^14^), sleep (e.g., the National Sleep Foundation^15^), and healthy eating (e.g., World Health Organization^16^), is substantial, affecting millions of individuals^13,17,18^. This requires implementation of population-based interventions that are both cost-effective and practical when applied on a large scale, including those that support individuals. The widespread availability of the internet and the use of websites and smartphone applications have resulted in a growing interest in e- and m-Health interventions to promote healthy behaviours^19^. These interventions leverage digital technologies such as smartphones, apps, wearable activity trackers, and web-based programs to deliver health-related information and support to individuals^19^. By utilising the ubiquity of digital technologies, including smartphones, wearables, and online platforms, these interventions engage individuals in health-related activities, offer tailored interventions, and facilitate continuous monitoring and feedback. The advantages of these technologies in promoting behaviour change include accessibility, individualisation, real-time feedback, and potential scalability^20^. Among the health behaviours targeted by these interventions, physical activity, sedentary behaviour, healthy eating and sleep, have emerged as key areas for promoting overall health and preventing chronic diseases^6–8^.

E- and m-Health interventions often employ behaviour change techniques such as goal setting, self-monitoring, feedback, and social support to encourage individuals to adopt and sustain healthy behaviours^19^. They frequently incorporate elements such as gamification, personalised messaging, machine learning, or other strategies to enhance engagement and motivation^19^. To gain a better understanding of the effectiveness of e- and m-Health interventions, numerous studies have been conducted in recent years to assess their impact on health behaviours, and the findings have been summarised in numerous previous systematic reviews^21–27^. Yet, these previous systematic reviews have varied widely, including different types of e- and m-Health interventions (e.g., SMS only^21^, or mobile apps only^22^), focussed on a specific behaviour only (e.g., physical activity^23^ or diet only^24^), or focussed on specific populations (e.g., breast cancer^25^, cardiovascular disease^26^, older adults^27^). This meta-analysis of meta-analyses aims to consolidate and amalgamate the existing knowledge in this field. Specifically, this meta-meta-analysis aims to:

1. Synthesise the current evidence regarding the effectiveness of e- and m-Health interventions to improve physical activity, sedentary behaviour, diet and sleep using meta-analysis.
2. Determine whether intervention effects differ based on participant characteristics (age, sex); population; e- and m-Health intervention approach (mobile apps, web-based, SMS, mixed [which included combinations of at least three of the other modes]); and AMSTAR-2 quality rating score.

## Results

**Fig. 1:**
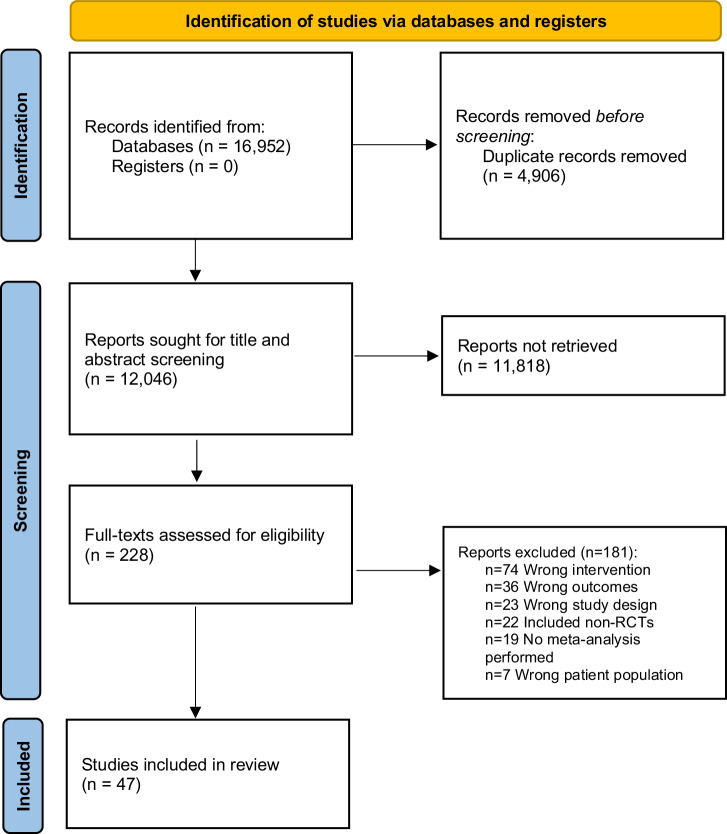
PRISMA flow chart. This diagram illustrates the selection process of studies for the systematic review, from initial identification through final inclusion.

Of the 16,952 records identified, 47 were eligible (see Fig. 1 for PRISMA flowchart including reasons for exclusions). They included 507 unique RCTs and the Corrected Covered Area (CCA) was 0.5%, indicating slight overlap. An overview of all reviews’ characteristics is shown in Supplementary Table 1. There was a total of *n* = 206,873 participants. Mean participant age ranged between 20.4 and 82.0 years, and most reviews (*n* = 42, 89%) involved a mix of female and male participants. The populations that were most commonly examined were the general population (*n* = 9), adults with overweight or obesity (*n* = 9), a mix of various chronic diseases (*n* = 5), survivors of cancer (*n* = 4) and other (*n* = 20, see Supplementary Table 1 for full description of populations). Fifteen reviews (31.9%; consisting of 287 component RCTs and 79,632 participants) specifically focused on physical activity e- and m-Health interventions, three (6.4%; consisting of 68 component RCTs and 17,679 participants) on diet interventions, four (8.5%; consisting of 81 component RCTs and 27,978 participants) on sleep interventions and 25 (53.2%; consisting of 471 component RCTs and 109,227 participants) evaluated combined behaviours. Description of the types of e- and m-Health interventions that were included in each review are shown in Supplementary Table 1 and included mobile apps, web-based and SMS interventions. Most reviews (*n* = 27, 57.4%) had a critically low AMSTAR-2 score (low: *n* = 15, 31.9%; moderate: *n* = 1, 2.1%; high: *n* = 4, 8.5%), Supplementary Table 2). For mean differences (MD), there was sufficient data to perform meta-analyses for: daily steps (steps/day), moderate-to-vigorous physical activity (MVPA, min/week), total physical activity (min/week), sedentary behaviour (min/week), fruit and vegetable consumption (serving/day), energy intake (kcal/day), saturated fat consumption (grams/day) and weight (kilograms). For standardised mean differences (SMD), there was sufficient data to perform meta-analyses on the following outcomes: daily steps, MVPA, total physical activity, sedentary behaviour, fruit and vegetable consumption, sleep quality, insomnia severity and weight. Visual analyses of funnel plots for meta-analyses with at least 10 studies included showed no evidence of publication bias (total physical activity: Supplementary Fig. 1; weight: Supplementary Fig. 2).

**Fig. 2:**
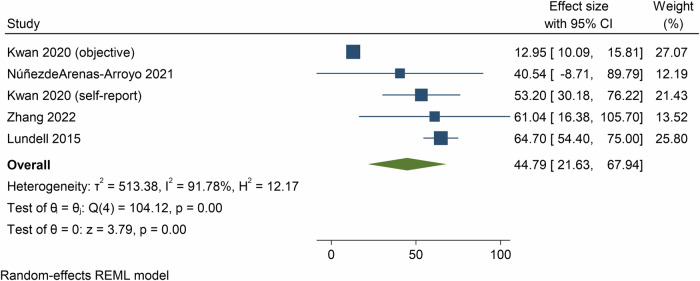
Forest plot showing the mean differences in total physical activity (minutes per week) resulting from eHealth and mHealth interventions compared to control groups. CI confidence interval.

Pooled analysis of 5 meta-analyses showed that the effect of e- and m-Health interventions on total physical activity was 44.8 min/week (95% CI = 21.6, 67.9, *p* < 0.01; *n* = 5 meta-analyses, 73 component RCTs, 18,608 participants, *I*^2^ = 91.8%, Fig. 2) at post-intervention, and 101.8 min/week (95% CI = −15.8, 219.3, *p* = 0.09; *n* = 1 meta-analysis, 8 component RCTs, 1495 participants, *I*^2^ = 0.0%, Supplementary Fig. 3) at follow-up.

Meta-analyses based on SMDs showed a medium effect of e- and m-Health interventions on total physical activity at post-intervention (SMD = 0.28, 95% CI = 0.21, 0.35, *p* < 0.01; 16 meta-analyses, 263 component RCTs, 62,377 participants, *I*^2^ = 51.2%, Supplementary Fig. 4) and follow-up (SMD = 0.29, 95% CI = 0.20, 0.38, *p* < 0.01; 2 meta-analyses, 25 component RCTs, *I*^2^ = 0.0%, 3566 participants, Supplementary Fig. 5).

**Fig. 3:**
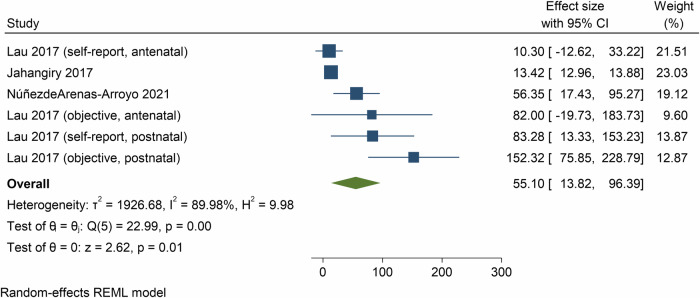
Forest plot showing the mean differences in moderate-to-vigorous physical activity (minutes per week) resulting from eHealth and mHealth interventions compared to control groups. CI confidence interval.

Pooled analysis of 3 meta-analyses showed the effect of e- and m-Health interventions on MVPA was 55.1 min/week (95% CI = 13.8, 96.4, *p* = 0.01; *n* = 3 meta-analyses, 54 component RCTs, 39,057 participants, *I*^2^ = 90.0%, Fig. 3) at post-intervention, and 50.2 min/week (95% CI = −26.2, 126.6, *p* = 0.20; *n* = 1 meta-analysis, 14 component RCTs, 3169 participants, *I*^2^ = 94.4%, Supplementary Fig. 6) at follow-up.

Meta-analyses based on SMDs showed a medium effect of e- and m-Health interventions on MVPA at post-intervention (SMD = 0.22, 95% CI = 0.14, 0.30, *p* < 0.01; 5 meta-analyses, 122 component RCTs, 20,801 participants, *I*^2^ = 0.0%, Supplementary Fig. 7).

**Fig. 4:**
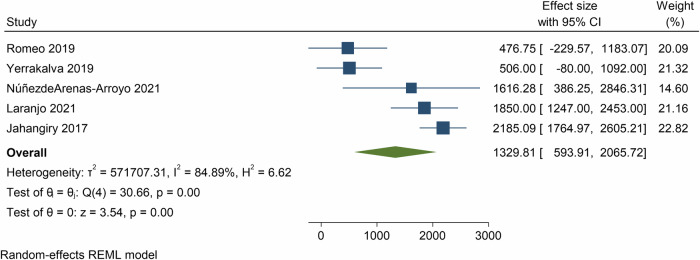
Forest plot showing the mean differences in daily steps resulting from eHealth and mHealth interventions compared to control groups. CI confidence interval.

Pooled analysis of 5 meta-analyses showed that the effect of e- and m-Health interventions was 1329 steps per day (95% CI = 593.9, 2065.7, *p* < 0.01; 89 component RCTs, 45,568 participants, *I*^2^ = 84.9%, Fig. 4) at post-intervention and 752 steps per day (95% CI = −147, 1651, *p* = 0.10; *n* = 1 meta-analysis, 5 component RCTs, 486 participants, *I*^2^ = 0.0%, Supplementary Fig. 8) at follow-up.

Meta-analysis based on SMD showed a medium effect for e- and m-Health interventions for increasing daily steps at post-intervention (SMD = 0.46, 95% CI = 0.29, 0.62, *p* < 0.01; 6 meta-analyses, 151 component RCTs, 30,474 participants, *I*^2^ = 28.5%, Supplementary Fig. 9). The grade of recommendation for all physical activity outcomes was: A) consistent level 1 studies.

**Fig. 5:**
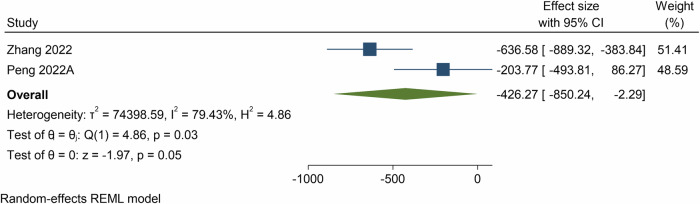
Forest plot showing the mean differences in sedentary behaviour (minutes per week) resulting from eHealth and mHealth interventions compared to control groups. CI confidence interval.

Meta-analyses based on MDs showed the effect of e- and m-Health interventions on sedentary behaviour was −426.3 min/week (95% CI = −850.2, −2.3, *p* = 0.05; *n* = 2 meta-analyses; 17 component RCTs, 2622 participants, *I*^2^ = 79.4%, Fig. 5) at post-intervention. The grade of recommendation for sedentary behaviour was: A) consistent level 1 studies.

**Fig. 6:**
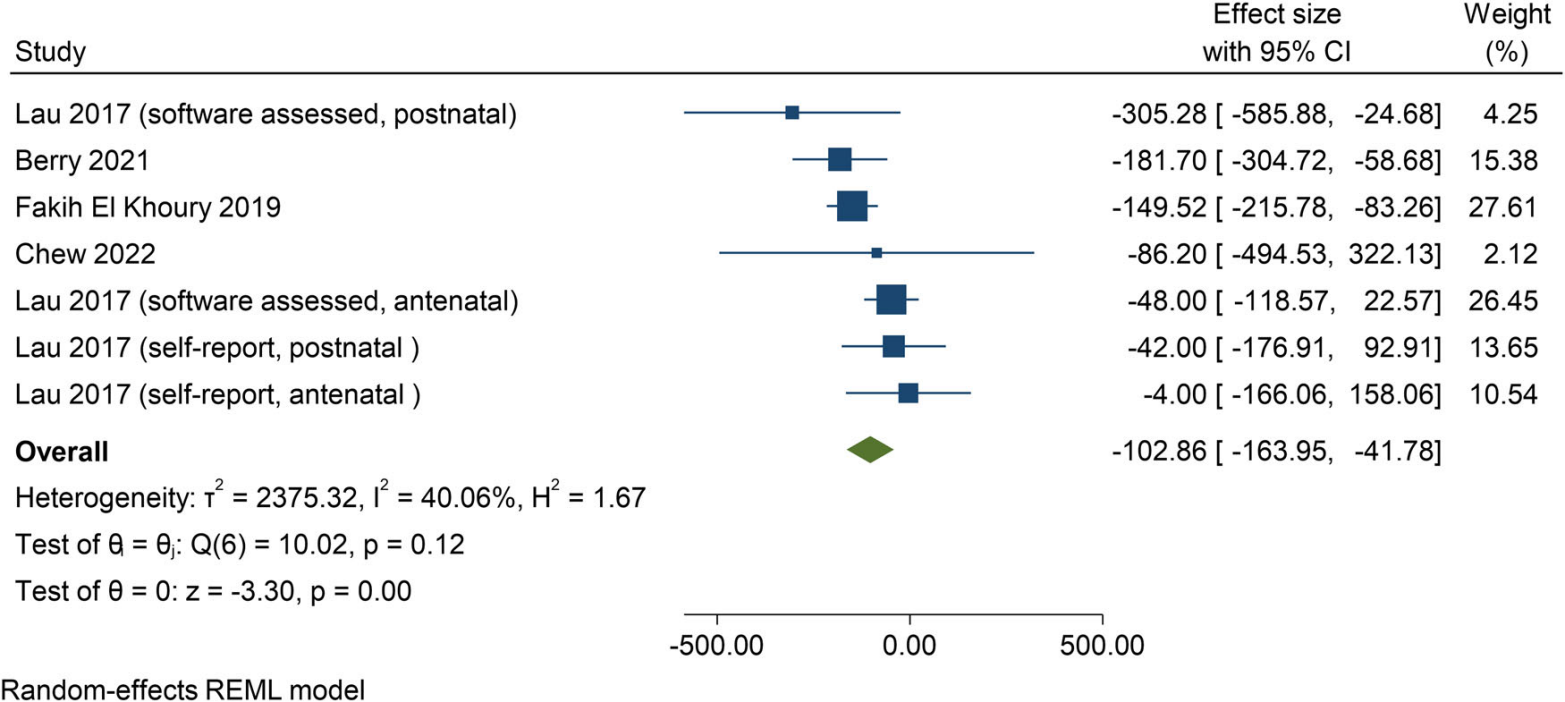
Forest plot showing the mean differences in daily energy intake (kilocalories per day) resulting from eHealth and mHealth interventions compared to control groups. CI confidence interval.

Meta-analyses based on MDs showed the effect of e- and m-Health interventions on energy intake was −102.9 kcals/day (95% CI = −164.0, −41.8, *p* < 0.01; *n* = 4 meta-analyses, 63 component RCTs, 9102 participants, *I*^2^ = 40.1%, Fig. 6) at post-intervention.

Pooled analysis of 3 meta-analyses showed the effect of e- and m-Health interventions on increasing fruit and vegetable consumption was 0.57 servings/day (95% CI = 0.11, 1.02, *p* = 0.01; 75 component RCTs, 12,375 participants, *I*^2^ = 55.8%, Supplementary Fig. 10) at post-intervention.

Meta-analyses based on SMD showed a medium effect of e- and m-Health interventions on increasing fruit and vegetable consumption (SMD = 0.23, 95% CI = 0.13, 0.33, *p* < 0.01; 2 meta-analyses, 29 component RCTs, 10346 participants, *I*^2^ = 0.0%, Supplementary Fig. 11) at post-intervention.

Meta-analyses based on MDs showed the effect of e- and m-Health interventions on saturated fat consumption was −5.5 grams/day (95% CI = −17.5, 6.4, *p* = 0.36; *n* = 1 meta-analysis, 14 component RCTs, 3169 participants, *I*^2^ = 69.2%, Supplementary Fig. 12) at post-intervention. The grade of recommendation for diet outcomes was: A) consistent level 1 studies.

Pooled analysis of 2 meta-analyses showed a large effect of e- and m-Health interventions on increasing sleep quality (SMD = 0.56, 95% CI = 0.40, 0.72, *p* < 0.01; 44 component RCTs, 15,100 participants, *I*^2^ = 0.0%, Supplementary Fig. 13) and reducing insomnia severity (SMD = −0.90, 95% CI = −1.14, −0.65, *p* < 0.01; 59 component RCTs, 23,713 participants, *I*^2^ = 78.7%, Supplementary Fig. 14) at post-intervention. The grade of recommendation for all sleep outcomes was: A) consistent level 1 studies.

Pooled analyses showed that the reduction in bodyweight was −1.89 kg (95% CI = −2.42, −1.36, *p* < 0.01; 11 meta-analyses, 175 component RCTs, 26468 participants, *I*^2^ = 76.4%, Supplementary Fig. 15) at post-intervention. Meta-analysis based on SMD showed a small effect of e- and m-Health interventions for reducing bodyweight (SMD = 0.16, 95% CI = −0.46, 0.15, *p* = 0.31; 152 component RCTs, 23457 participants, *I*^2^ = 93.5%, Supplementary Fig. 16) at post-intervention. The grade of recommendation for all weight-related outcomes was: A) consistent level 1 studies.

There were no subgroup differences based on age, sex, population, intervention type or AMSTAR-2 score for total physical activity based on MD and SMD (all *p* > 0.05, Supplementary Figs. 17, 18).

There were no subgroup differences based on age, sex and population for MVPA based on MD (all *p* > 0.05, Supplementary Fig. 19). There was a significant subgroup effect for intervention type (Q_b_(2) = 8.64, *p* = 0.01) and AMSTAR-2 score (Q_b_(2) = 8.17, *p* = 0.02), with mixed interventions showing an increase of 74.8 mins/week, mobile and web-based interventions showing an increase of 56.3 mins/week and web-based only interventions showing an increase of 13.4 min/week (note, there were an insufficient number of studies in the other categories). Interventions with a critically low, low and high AMSTAR-2 score showed an increase of 13.4 mins/week, 56.4 mins/week and 74.8 mins/week, respectively. There were no subgroup differences (all *p* > 0.05, Supplementary Fig. 20) for MVPA based on SMD.

Subgroup analyses for steps based on MD showed that the effects differed by population (Q_b_(1) = 9.92, *p* < 0.01) and intervention type (Q_b_(2) = 10.00, *p* = 0.01, Supplementary Fig. 21). Larger effects were observed in participants with overweight and obesity, compared with the general population (MD = 2185.1 steps/day and 1006.8 steps/day, respectively), and greater effects with web-based only interventions, compared with mobile plus web-based interventions, and mobile app only interventions (MD = 2185.1 steps/day, 1616.3 steps/day and 911.3 steps/day, respectively). For the effects based on SMD, there were no subgroup differences (all *p* > 0.05, Supplementary Fig, 22).

There were no subgroup effects of sex and AMSTAR-2 score on fruit and vegetable consumption (Supplementary Fig. 23). For age, a larger increase in fruit and vegetable consumption was observed for those aged 50 or less (2 servings per day), than those over 50 (0.6 servings per day) and studies where age was not reported (0.3 servings per day; (Q_b_(2) = 7.01, *p* = 0.03). A greater increase in fruit and vegetable consumption was observed among individuals with overweight or obesity (2 servings per day) than individuals with various chronic diseases (0.4 servings per day; (Q_b_(1) = 4.13, *p* = 0.04). A greater increase in fruit and vegetable consumption was shown in mixed interventions (2 servings/day) than mobile apps, SMS and web-based interventions (0.6 servings/day) and mobile apps only (0.27 servings/day; (Q_b_(2) = 7.01, *p* = 0.03).

There were no subgroup effects for sex, population or AMSTAR-2 score for energy consumption (Supplementary Fig. 24). A greater reduction in energy intake was observed in studies where age was not reported (−156.8 kcals/day) compared with those aged 50 or less (−52.8 kcals/day (Q_b_(1) = 6.29, *p* = 0.01). A greater reduction in energy consumption was observed following mobile and web-based interventions (−181.7 kcals/day) than mobile apps only (−147.9 kcals/day) and mixed interventions (−52.1 kcals/day; (Q_b_(2) = 6.43, *p* = 0.04; note, there were an insufficient number of studies in the other categories).

There were no significant subgroup effects for weight based on MD and SMD (Supplementary Figs. 25, 26).

## Discussion

This umbrella review set out to consolidate and assess the cumulative evidence on the impact of e- and m-Health interventions on 24-hour movement behaviours and diet. We identified 47 meta-analyses, comprising 507 RCTs, with a total of 206,873 participants. The combined findings from meta-analyses revealed that e- and m-Health interventions resulted in an increase of 1329 steps per day, an increase of 55 min/week in MVPA, an increase of 45 min/week in total physical activity, a decrease of 7 hours/week in sedentary behaviour, an increase of 0.6 servings/day in fruit and vegetable consumption, a reduction of 103 kcals/day in energy intake, a decrease of 6 g/day in saturated fat consumption, and a decrease of 1.9 kg in body weight. Meta-analyses also demonstrated large effect size improvements in sleep quality and insomnia severity. Taken together, the results underscore the effectiveness of e- and m-Health interventions across various health behaviours, intervention approaches, and age and health populations.

Physical activity emerged as a primary focus in the included systematic reviews and meta-analyses, with results graded as Grade A, demonstrating consistent level 1 studies. In comparison to a recent umbrella review of wearable activity trackers^28^, the e- and m-Health interventions in our review showed a similar improvement in daily steps ( + 1330 steps compared to +1800 steps in Ferguson et al.^28^) and MVPA (+8 min per day compared to +6 min in Ferguson et al.^28^). It is important to emphasise that these observed changes in physical activity were not only statistically significant but also clinically meaningful, indicating a substantial impact on individuals’ overall health and well-being. Moreover, our study provides some indication of the sustainability of these improvements during long-term follow-up assessments (e.g., for total physical activity and MVPA), suggesting that the positive effects of these interventions may persist after the intervention has concluded. This indication of promising long-term maintenance underscores the benefits of e- and m-Health interventions in promoting physical activity.

Dietary intake emerged as the second most extensively investigated health behaviour, and the findings from meta-analyses indicate that e- and m-Health interventions have small-to-moderate improvements across various dietary outcomes. These interventions have proven effective in promoting increased consumption of fruits and vegetables while simultaneously reducing saturated fat and energy intake. Furthermore, enhancing nutritional intake stands as a pivotal component of comprehensive treatment programs aimed at mitigating the risk of cardiovascular disease and diabetes^29,30^. Although a decrease in daily energy intake is associated with improved weight status, adiposity, and metabolic syndrome risk reduction^30^, it is worth noting that the average reduction of 103 kcals/day, as highlighted in this review, may not meet the >5% to ≥10% recommended energy intake reduction threshold. Nonetheless, this finding is in line with the review’s identification of a modest 1.9 kg weight loss. Even such a seemingly modest weight reduction can yield substantial health benefits, particularly considering that a 5–10% reduction in total body weight is often advised for noticeable health enhancements^31^. In this context, a 1.9 kg decrease can represent a significant step forward, especially for individuals embarking on their weight loss journey.

This review underscored that sedentary behaviour and sleep, despite their significant implications for health, have been less emphasised in e- and m-Health intervention research compared with physical activity and dietary behaviours. For sedentary behaviour, our study revealed a notable reduction of 427 min per week, which translates to approximately 1 h less per day. As recent literature suggests, the focus on sedentary behaviour interventions is gaining traction^32^. Such notable reductions underscore the health potential of these interventions^33^ and emphasise their contribution in guiding individuals towards meeting established activity and sedentary behaviour guidelines^34^. Regarding sleep, our findings highlight the efficacy of e- and m-Health platforms in fostering healthier sleep habits. Although sleep interventions traditionally catered to specific pathologies like sleep apnoea^35^, a significant proportion of the general population, estimated at around 30%, experiences sleep difficulties^36^, pointing to an unmet need for broader interventions. E- and m-Health interventions, given their broad reach and adaptability, emerge as a potent solution for providing comprehensive sleep programs for the general public.

The subgroup analyses revealed variability in the effectiveness of digital health interventions across certain participant and intervention factors, although overall effects were largely consistent. More pronounced differences emerged for specific outcomes like MVPA, steps, and fruit/vegetable consumption; for example, larger benefits were observed in those with overweight/obesity compared to the general population for steps and produce intake. Web-based platforms also outperformed apps and mixed approaches for steps and MVPA. However, intervention effects were generally similar across subgroups for total physical activity, weight, and energy intake, with a few exceptions like greater energy reduction in younger adults. While digital interventions appear broadly effective overall, tailoring specific platform types and delivery modes to particular populations may further optimise impacts on outcomes like MVPA and healthy eating. Carefully considering intervention design and individualised intervention components may be worthwhile to maximise effectiveness.

This study boasts several key strengths. Foremost, its rigorous methodology adheres to the PRISMA 2020 guidelines, and the comprehensive search strategy produced an extensive dataset of 47 systematic reviews and meta-analyses, encapsulating 507 unique RCTs and encompassing 206,873 participants. The minimal redundancy evident from a CCA of 0.5% ensures our findings are drawn from a diverse and predominantly independent evidence base, lending substantial weight to the generalisability of our conclusions. By pooling data from multiple meta-analyses, the meta-meta-analytical approach provides a thorough synthesis of the current evidence, strengthening the reliability of the effects reported. The wide-ranging populations and interventions studied further expand the understanding of the versatility and relevance of e- and m-Health interventions across diverse contexts and demographics.

Despite its numerous strengths, the study is not without limitations. A significant concern is the quality of the reviews included, with a majority (57.4%) having a critically low AMSTAR-2 score, which may impact the overall trustworthiness of the findings. Additionally, the meta-analysis, by its nature, could obscure heterogeneity in individual studies or mask specific nuances that might be of significance. For example, interventions focusing on populations with lower initial levels of physical activity or fruit/vegetable consumption, such as individuals with obesity, may have greater effects in promoting these behaviours. However, our ability to investigate baseline differences between subgroups is constrained due to the synthesis of aggregate effect estimates from systematic reviews. The finding that the included reviews relied on largely distinct sets of RCTs suggests there is heterogeneity in the literature and potential subgroup differences between studies that we were unable to assess. Moving forward, we suggest further examination of potential clinical and methodological subgroups that may account for differences between reviews. Carefully delineating patient populations, interventions, comparators, and outcomes in included trials may reveal explanations for the lack of overlap and inconsistencies between past syntheses. Finally, the general challenges associated with e- and m-Health studies, such as technological disparities among participants or rapidly evolving technology landscapes, might also affect the results’ applicability over time.

The observed variability in intervention effectiveness across different populations and intervention types from the subgroup analyses can be interpreted in several ways. The lack of significant differences in many subgroups might suggest that more complex and expensive interventions don’t necessarily outperform simpler e- and m-Health strategies. This is encouraging as it hints at the potential for broader, cost-effective dissemination of digital health tools, reaching more people without a heavy resource investment. However, as the field of digital health interventions progresses, there’s likely untapped potential for individualised, tailored approaches. Such bespoke interventions could cater to the distinct needs and preferences of specific user groups, potentially boosting adherence and enhancing outcomes. We also recognise that technology access and cultural factors related to technology use may vary across populations and should be considered when interpreting these findings. While this review provides evidence that digital health interventions can be broadly effective, it does not explore potential differences in impacts for specific cultural or demographic groups with differing technology access or tech usage patterns. Further research into population and cultural differences would provide valuable context about whether effectiveness may differ based on the population or region. Future research should also prioritise in-depth exploration of adherence to e- and mHealth interventions, recognising that while these interventions demonstrate efficacy when adhered to, suboptimal adherence remains a significant challenge, warranting investigation into the valuable insights that detailed adherence data can provide.

Overall, the findings of this study underscore the potential of e- and m-Health interventions as transformative tools in the promotion of healthier behaviours across various domains, from physical activity, sedentary behaviour and sleep to dietary habits. Future research should delve deeper into understanding which specific elements of e- and m-Health interventions resonate most effectively with different user profiles. Given the inconsistency in subgroup effects, it would be beneficial to explore the underlying mechanisms that make certain interventions more effective for specific groups. Additionally, with the rapid evolution of technology and user interfaces, studies should remain abreast of the latest digital trends to ensure interventions remain engaging and effective. In particular, conversational agents (i.e., chatbots) are emerging digital health support tools, and a recent review of experimental studies suggesting they hold considerable promise for intervening on physical activity and diet^37^. Future systematic reviews and meta-analyses should also provide detailed reporting of behaviour change techniques utilised in individual trials. This information is crucial for identifying effective intervention components and informing the development of future interventions. There’s also an opportunity to investigate the long-term sustainability of behaviour changes induced by e- and m-Health interventions. It would be pivotal to ascertain not just the immediate impact but also the longevity of such changes in real-world scenarios, ultimately driving the design of more sustainable and impactful digital health solutions.

This review highlights the promise of e- and m-Health interventions across various health behaviours. For designers or developers, a range of different e- and m-Health interventions can generally be effective, however opportunities exist for more personalised approaches to optimise engagement. Public health researchers should conduct higher-quality trials examining optimal designs for specific populations. Healthcare providers can recommend appropriate e- and m-Health interventions to promote lifestyle change in patients. The general public can benefit from reputable, evidence-based digital resources that increase physical activity, improve diet, reduce sedentary time, and address sleep issues, even with modest sustained changes. Collectively, these findings underscore the potential of e- and m-Health solutions but also indicate needs for tailoring, sustained adherence strategies, and further study of long-term impacts across user groups.

In conclusion, this rigorous meta-meta-analysis offers a promising snapshot of the efficacy and potential of e- and m-Health interventions in shaping healthier behaviours across various populations. With consistent findings pointing to tangible improvements in physical activity, dietary habits, sleep, and sedentary behaviour, e- and m-Health tools stand as an important approach in an ever-evolving health landscape. Importantly, while the immediate impacts are evident, the longevity and sustainability of these interventions in real-world contexts remain paramount for future investigations. As technology continues its inexorable march forward, it will be crucial for health interventions to adapt, innovate, and maintain relevance. This review not only underscores the benefits of such interventions but also sets a roadmap for future research – emphasising the need for personalised, adaptable, and enduring digital health strategies. The present results affirm the potential of e- and m-Health tools in driving meaningful and lasting changes in health behaviours, benefiting a diverse array of populations across the globe.

## Methods

The protocol was pre-registered on PROSPERO (Registration ID: CRD42023418570), and the reporting of results adheres to the PRISMA 2020 guidelines.

We used the Population, Intervention, Comparison, Outcomes, and Study Type (PICOS) framework to formulate the inclusion criteria in the following manner: Population: any adult population (aged ≥18 years). Reviews that included RCTs involving children or adolescents were excluded. Intervention: E- and m-Health interventions targeting physical activity, sedentary behaviour, diet or sleep. For inclusion, the systematic reviews had to be exclusively focused on e- or m-Health interventions, and ≥75% of the included RCTs in these reviews were required to be delivered solely through e- or m-Health interventions. Reviews were excluded if an eligible e- or m-Health intervention was combined with an ineligible intervention (e.g., physical activity plus medications), or if 25% or more of the included RCTs involved an ineligible intervention. Reviews were also excluded if they were focussed on wearable activity trackers to prevent overlap with a recently published umbrella review^28^. Comparator: Reviews were eligible if they compared an eligible e- or m-Health intervention (physical activity, sedentary behaviour, diet or sleep) arm to no intervention, including waitlist, usual care, a sham intervention or an equal attention non-physical activity, sedentary behaviour, diet or sleep intervention arm. Outcomes: The primary outcomes were any outcomes related to physical activity, sedentary behaviour, diet and sleep. Secondary outcomes were outcomes related to weight and adiposity. Study type: Systematic reviews with meta-analyses of RCTs only, that included meta-analyses of at least one of the outcomes of interest.

Nine databases were searched (CINAHL, the Cochrane Library, Embase via OVID, MEDLINE via OVID, Emcare via OVID, ProQuest central, ProQuest Nursing and Allied Health Source, PsycINFO, Scopus) using subject heading, keyword and MeSH term searches for “e-Health”, “m-Health”, “digital health”, “technology-based”, “physical activity”, “sedentary behaviour”, “sleep”, “healthy eating”, “diet”, “nutrition” “systematic review” and “meta-analysis” (see Supplementary Table 3 for the full search strategy). Database searches were limited to peer-reviewed journal articles published in English-language prior to 1 June 2023.

The search results were imported into EndNote x9 (from Clarivate, Philadelphia, PA) to eliminate duplicates and transferred to Covidence (developed by Veritas Health Innovation, Melbourne, Australia). Two separate reviewers conducted title/abstract and full-text screening, data extraction, and quality and risk of bias assessment. Data extraction was performed using a standardised extraction form. Disagreements were resolved through discussion. The quality and risk of bias assessment was performed by two independent reviewers in duplicate using the AMSTAR-2 tool, with ratings categorised as “high”, “moderate”, “low”, or “critically low” confidence^38^.

The evaluation of overlap among the RCTs in the included reviews was conducted using the CCA. The following equation was used: CCA = (N - r) ÷ (r × c) - r, where N is the total number of RCTs included across all reviews (including duplicates), r is the total number of unique RCTs (excluding duplicates), and c is the number of reviews included in the analysis^39^. The following categories were used: 0–5% denoted as “slight,” 6–10% as “moderate,” 11–15% as “high,” and >15% as “very high” overlap^39^. To assess publication bias, funnel plots were created, and the presence of asymmetries or missing data sections was visually examined for meta-analyses with at least 10 studies included^40^.

Meta-analyses of the outcomes of interest were performed by pooling the effect sizes and 95% confidence intervals (CIs) reported in each meta-analysis, using a random effects model. Results of all meta-analyses were displayed visually using forest plots. Separate meta-analyses were performed for standardised effect sizes (e.g., standardised mean difference, SMD) and unstandardised effect sizes (e.g., mean difference, MD), and the meta-analysed effect sizes (SMDs or MDs) were reported with 95% CIs. Subgroup analyses were performed for age (50 years or less, over 50), sex (female, male, not reported), population (general population, chronic disease), intervention type (mobile apps, web-based, SMS, mixed [which included combinations of at least three of the other modes]) and AMSTAR-2 (critically low, low, moderate, high) if more than 1 eligible meta-analysis was included in at least 2 of the groups. The I^2^ statistic was used to quantify the proportion of the overall outcome attributed to variability^40^. The following values were used to determine level of heterogeneity: I^2^ = 0-25%: low heterogeneity; I^2^ ≥ 25-50%: moderate heterogeneity; I^2^ ≥ 75-100%: high heterogeneity^40^. The following classifications for the magnitude of effect were used: less than 0.20 = small effect; 0.20 to 0.50 = medium effect; and greater than 0.50 = large effect^41^. A *p*-value of <0.05 was considered statistically significant. All meta-analyses were performed using Stata (v16, Stata Corp, College Station, TX, USA).

Levels of evidence and grades for recommendations^42^ were used to classify the overall level of evidence as grade A: consistent level 1 studies (i.e., systematic reviews of RCTs or individual RCTs); B: consistent level 2 (i.e., systematic reviews of cohort studies or individual cohort studies) or 3 studies (i.e., systematic reviews of case–control studies or individual case–control studies) or extrapolations from level 1 studies; C: level 4 studies (i.e., case series) or extrapolations from level 2 or 3 studies; or D: level 5 evidence (i.e., expert opinion without explicit critical appraisal) or troublingly inconsistent or inconclusive studies of any level^42^.

There were several variations to our pre-registered protocol. We were unable to complete the following planned subgroup analyses, due to insufficient data reported in the included meta-analyses: intervention based on a theoretical framework or theory (yes or no), number of behaviour change techniques, method of outcome assessment (self-report or objective), intervention length and time since follow-up (short-term versus longer term).

### Reporting summary

Further information on research design is available in the Nature Research Reporting Summary linked to this article.

### Supplementary information

Supplementary material

Reporting Summary

## Data Availability

B.S. has full access to all of the data in the study and takes responsibility for the integrity of the data and the accuracy of the data analysis. All study materials are available from the corresponding author upon reasonable request.

## Code availability

The codes used in the analysis of this study will be made available from the corresponding author upon reasonable request.

## References

[1] Hajat C, Stein E (2018). The global burden of multiple chronic conditions: A narrative review. Prev. Med. Rep..

[2] World Health Organisation. Noncommunicable diseases. 2023. https://www.who.int/news-room/fact-sheets/detail/noncommunicable-diseases (accessed September 22, 2023).

[3] Nyberg ST (2020). Association of healthy lifestyle with years lived without major chronic diseases. JAMA Intern Med..

[4] Booth FW, Roberts CK, Laye MJ (2012). Lack of exercise is a major cause of chronic diseases. Compr. Physiol..

[5] Adams ML, Grandpre J, Katz DL, Shenson D (2019). The impact of key modifiable risk factors on leading chronic conditions. Prev. Med..

[6] Kohl HW (2012). The pandemic of physical inactivity: global action for public health. Lancet.

[7] Stamatakis E (2019). Is the time right for quantitative public health guidelines on sitting? A narrative review of sedentary behaviour research paradigms and findings. Br. J. Sports Med.

[8] Endalifer ML, Diress G (2020). Epidemiology, predisposing factors, biomarkers, and prevention mechanism of obesity: a systematic review. J. Obes..

[9] Bloom, D. E. et al. The Global Economic Burden of Noncommunicable Diseases. Geneva: World Economic Forum (2011).

[10] Sequi-Dominguez I (2020). Effectiveness of mobile health interventions promoting physical activity and lifestyle interventions to reduce cardiovascular risk among individuals with metabolic syndrome: Systematic review and meta-analysis. J. Med Internet Res.

[11] Elwood P (2013). Healthy lifestyles reduce the incidence of chronic diseases and dementia: evidence from the Caerphilly cohort study. PLoS One.

[12] Park JH, Moon JH, Kim HJ, Kong MH, Oh YH (2020). Sedentary lifestyle: Overview of updated evidence of potential health risks. Korean J. Fam. Med.

[13] Alosaimi N, Sherar LB, Griffiths P, Pearson N (2023). Clustering of diet, physical activity and sedentary behaviour and related physical and mental health outcomes: a systematic review. BMC Public Health.

[14] Bull FC (2020). World Health Organization 2020 guidelines on physical activity and sedentary behaviour. Br. J. Sports Med.

[15] Hirshkowitz M (2015). National Sleep Foundation’s updated sleep duration recommendations: final report. Sleep. Health.

[16] World Health Organisation. Healthy diet. 2023. https://www.who.int/news-room/fact-sheets/detail/healthy-diet (accessed September 22, 2023).

[17] Du Y (2019). Trends in adherence to the physical activity guidelines for Americans for aerobic activity and time spent on sedentary behavior among US adults, 2007 to 2016. JAMA Netw. Open.

[18] Deslippe AL (2023). Barriers and facilitators to diet, physical activity and lifestyle behavior intervention adherence: A qualitative systematic review of the literature. Int J. Behav. Nutr. Phys. Act..

[19] Davis A, Sweigart R, Ellis R (2020). A systematic review of tailored mHealth interventions for physical activity promotion among adults. Transl. Behav. Med.

[20] Milat AJ, King L, Bauman AE, Redman S (2013). The concept of scalability: Increasing the scale and potential adoption of health promotion interventions into policy and practice. Health Promot Int.

[21] Smith DM, Duque L, Huffman JC, Healy BC, Celano CM (2020). Text message interventions for physical activity: A systematic review and meta-analysis. Am. J. Prev. Med.

[22] Schoeppe S (2016). Efficacy of interventions that use apps to improve diet, physical activity and sedentary behaviour: A systematic review. Int. J. Behav. Nutr. Phys. Act..

[23] Kwan RYC (2020). The effect of e-health interventions promoting physical activity in older people: A systematic review and meta-analysis. Eur. Rev. Aging Phys. Act..

[24] Robert C (2021). Effectiveness of ehealth nutritional interventions for middle-aged and older adults: Systematic review and meta-analysis. J. Med Internet Res.

[25] Dorri S, Asadi F, Olfatbakhsh A, Kazemi A (2020). A systematic review of electronic health (eHealth) interventions to improve physical activity in patients with breast cancer. Breast Cancer.

[26] Duff OM (2017). Behavior change techniques in physical activity eHealth interventions for people with cardiovascular disease: Systematic review. J. Med. Internet Res..

[27] Elavsky S, Knapova L, Klocek A, Smahel D (2019). Mobile health interventions for physical activity, sedentary behavior, and sleep in adults aged 50 years and older: A Systematic literature review. J. Aging Phys. Act..

[28] Ferguson T (2022). Effectiveness of wearable activity trackers to increase physical activity and improve health: A systematic review of systematic reviews and meta-analyses. Lancet Digit Health.

[29] Nilsson PM, Tuomilehto J, Rydén L (2020). The metabolic syndrome – What is it and how should it be managed?. Eur. J. Prev. Cardiol..

[30] Samson SL (2023). American association of clinical endocrinology consensus statement: Comprehensive type 2 diabetes management algorithm - 2023 update. Endocr. Pr..

[31] Wing RR (2011). Benefits of modest weight loss in improving cardiovascular risk factors in overweight and obese individuals with type 2 diabetes. Diab. Care.

[32] Young DR (2016). Sedentary behavior and cardiovascular morbidity and mortality: A science advisory from the American Heart Association. Circulation.

[33] Biswas A (2015). Sedentary time and its association with risk for disease incidence, mortality, and hospitalization in adults: A systematic review and meta-analysis. Ann. Intern Med.

[34] Tremblay MS (2017). Sedentary behavior research network (SBRN)–terminology consensus project process and outcome. Int. J. Behav. Nutr. Phys. Act..

[35] Gottlieb DJ, Punjabi NM (2020). Diagnosis and management of obstructive sleep apnea: A review. JAMA.

[36] Roth T (2007). Insomnia: definition, prevalence, etiology, and consequences. J. Clin. Sleep. Med..

[37] Singh B (2023). Systematic review and meta-analysis of the effectiveness of chatbots on lifestyle behaviours. NPJ Digit Med..

[38] Shea BJ (2017). AMSTAR 2: a critical appraisal tool for systematic reviews that include randomised or non-randomised studies of healthcare interventions, or both. BMJ.

[39] Hennessy EA, Johnson BT (2020). Examining overlap of included studies in meta-reviews: Guidance for using the corrected covered area index. Res. Synth. Methods.

[40] Deeks, J. J., Higgins, J. P. T., Altman, D. G. on behalf of the Cochrane Statistical Methods G. Analysing data and undertaking meta-analyses. Cochrane Handbook for Systematic Reviews of Interventions (2nd Edition). (2019).

[41] Lakens D (2013). Calculating and reporting effect sizes to facilitate cumulative science: a practical primer for t-tests and ANOVAs. Front Psychol..

[42] Oxford Centre for Evidence-Based Medicine. Oxford Centre for Evidence-Based Medicine: Levels of Evidence, March 2009. (2009). https://www.cebm.ox.ac.uk/resources/levels-of-evidence/oxford-centre-for-evidence-based-medicine-levels-of-evidence-march-2009 (accessed September 22, 2023).

