# Supplementary material for: A systematic umbrella review and meta-meta-analysis of eHealth and mHealth interventions for improving lifestyle behaviours
Source: NPJ Digit Med. 2024 Jul 5;7:179. doi: 10.1038/s41746-024-01172-y (PMC11226451; doi:10.1038/s41746-024-01172-y)
Supplement: Supplementary file 1 — Supplementary material [file 41746_2024_1172_MOESM1_ESM.pdf]

Contents

Supplementary Table 1. Overview of all included studies. 3

Supplementary Table 2. AMSTAR 2 quality appraisal of reviews. 10

Supplementary Figure 1. Funnel plot for total physical activity (standardised mean difference).. 12

Supplementary Figure 2. Funnell plot for weight (mean difference) 13

Supplementary Figure 3. Meta-analyses of the effects of E- and M-health interventions on total physical activity at follow-up (mean differences; mins/week) 14

Supplementary Figure 4. Meta-analysis of the effects of E- and M-health interventions on total physical activity outcomes, standardised mean differences. 15

Supplementary Figure 5. Meta-analyses of the effects of E- and M-health interventions on total physical activity at follow-up, standardised mean differences. 16

Supplementary Figure 6. Meta-analyses of the effects of E- and M-health interventions on moderate-to-vigorous physical activity at follow-up (mean differences; mins/week). 17

Supplementary Figure 7. Meta-analyses of the effects of E- and M-health interventions on moderate-to-vigorous physical activity, standardised mean differences. 18

Supplementary Figure 8. Meta-analyses of the effects of E- and M-health interventions on steps per day at follow-up (mean differences; steps/day). 19

Supplementary Figure 9. Meta-analyses of the effects of E- and M-health interventions on daily steps, standardised mean differences. 20

Supplementary Figure 10. Meta-analyses of the effects of E- and M-health interventions on fruit and vegetable consumption (mean differences; servings/day)...................................... 21

Supplementary Figure 11. Meta-analysis of the effects of E- and M-health interventions on fruit and vegetable consumption (standardised mean differences). 22

Supplementary Figure 12. Meta-analyses of the effects of E- and M-health interventions on saturated fat consumption (mean differences; grams/day). 23

Supplementary Figure 13. Meta-analysis of the effects of E- and M-health interventions on sleep quality (standardised mean differences). 24

Supplementary Figure 14. Meta-analysis of the effects of E- and M-health interventions on insomnia severity (standardised mean differences). 25

Supplementary Figure 15. Meta-analyses of the effects of E- and M-health interventions on weight change (mean differences; kgs). 26

Supplementary Figure 16. Meta-analysis of the effects of E- and M-health interventions on weight change (standardised mean differences). 27

Supplementary Figure 17. Subgroup analyses for total PA (mean difference, min/week). 28

Supplementary Figure 18. Subgroup analyses for total PA (standardised mean difference). . 29

Supplementary Figure 19. Subgroup analyses for MVPA (mean difference, min/week) 30

Supplementary Figure 20. Subgroup analyses for MVPA (standardised mean difference). .. 31

Supplementary Figure 21. Subgroup analyses for steps (mean difference, steps/day). 32
Supplementary Figure 22. Subgroup analyses for steps (standardised mean differences). 33
Supplementary Figure 23. Subgroup analyses for on fruit and vegetable consumption (mean difference, servings/day) 34
Supplementary Figure 24. Subgroup analyses for energy intake (mean differences, kcals/day). 35
Supplementary Figure 25. Subgroup analyses for weight (standardised mean difference). ... 36
Supplementary Figure 26. Subgroup analyses for weight (mean difference, kg) 37
Supplementary Table 3. Medline search strategy and terms 38

Supplementary Table 1. Overview of all included studies.

Author, year, country	Studies (k=)	Total sample (n=)	Population or condition; Gender; Age mean±SD or range (years)	Target behaviours (n= studies)	Intervention details	Relevant outcomes of interest
Alhussein, 2022, United Arab Emirates	32	14,235	Adults with osteoporosis<br>Females & males<br>Age range: 18 to 80	Physical activity & diet: n=11<br>Lifestyle & other: n=20<br>Sleep: n=1	Mobile app only n=32	Physical activity<br>Diet
Alsahli 2022. United Arab Emirates	6	484	Adults with Type-2 Diabetes<br>Females & males<br>Mean age range: 44.6 to 65.5	Physical activity: n=6	Text messaging alone n=1<br>Text message & pedometers n=3<br>Text message & educational CD n=1	Physical activity
Arambepola 2016, United Kingdon	15	NR	Adults with Type-2 Diabetes<br>Females & males<br>Age: NR	Physical activity & diet: n= 14<br>Diet alone: n=1	Text message n=12<br>Graphical information n=1<br>Text message via website n=1<br>Video messages n=1	Weight and adiposity-related outcomes
Barnett 2022, Australia	39	7,333	Adults with an established diet-related chronic disease<br>Females & males<br>Mean age range: 43.3 to 64.8	Diet: n=39	Internet-based: n=17<br>Mobile SMS: n=9<br>Mobile app: n=5<br>Mobile app with SMS: n=2<br>Internet-based & Mobile SMS: n=2<br>Internet-based & mobile app: n=1<br>Mobile interactive voice call: n=1<br>Electronic software: n=1<br>Internet-based, mobile app and SMS: n=1	Diet
Beleigoli 2019, Brazil	11	1525	Adults with overweight & obesity<br>Females & males<br>Age range: 18-65	Physical activity and diet: n=11	Website n=6<br>Mobile app n=2<br>Other/not-specified n=3	Weight
Berry 2021, United Kingdon	11	1190	Adults with overweight & obesity<br>Females & males<br>Age: NR	Physical activity: n=5<br>Diet: n=6	Website & phone calls n=3<br>Mobile app only n=2<br>Mobile app & phone calls n=1<br>Mobile app & website n=1<br>App & phone calls n=1<br>Website & email n=1<br>Other n=2	Weight<br>Diet
Bunting 2021, United Kingdom	5	518	Adults with musculoskeletal pain<br>Females & males<br>Age: NR	Physical activity: n=5	Website only n=2<br>Mobile app only n=1<br>Audiotape n=1<br>Video tape n=1	Physical activity
Chew 2022	16	2870	Adults with overweight & obesity<br>Females & males<br>Range mean age: 22.7-70.1	Physical activity & Diet: n=10<br>Diet: n=6	Smartphone Apps n=16	Weight

Author, year, country	Studies (k=)	Total sample (n=)	Population or condition; Gender; Age mean±SD or range (years)	Target behaviours (n= studies)	Intervention details	Relevant outcomes of interest
David 2022	44	24,692	General population<br>Female & males<br>Range of mean ages: 27.1-69.5	Physical activity & diet: n= 41<br>Diet: n=3	Text message n=24<br>App n=12<br>Text message & App n=8	Diet and adiposity-related outcomes, including blood pressure
Deng 2023	37	13,227	Adults with insomnia<br>Female & males<br>Range of mean ages: 24.6 to 58.6	Sleep: n=37	Computer-assisted n=13<br>Web & email combination n=1<br>Mobile & phone combination n=1<br>Phone-delivered n=4<br>Mobile & email combination n=1<br>Animated therapist guide with email/phone/text reminders n=6<br>Unguided self-help with reminders n=7 Mixed n=4	Sleep
Duan 2021	15	4725	Noncommunicable diseases<br>Female & males<br>Range of mean ages: 42.3-73.0	Physical activity & diet: n=15	Web-based + SMS: n=4<br>Web-based: n=3<br>SMS: n=2<br>Web-based + phone call: n=1<br>Mixed or other: n=5	Physical activity<br>Diet
Emmerson 2019	14	2156	Various health conditions (inc. incontinence, orthopaedic conditions, pulmonary, cardiac, voice disorders, traumatic injury, women's health, & neurological conditions).<br>Female & males<br>Range mean ages: 20 to 71.1	Physical activity: n=14	Audiotapes, videotapes, or DVDs: n=10<br>Mobile app: n=2<br>SMS: n=2	Physical activity
El Khoury 2019	22	1873	Adults with chronic diseases<br>Female & males<br>Adults 18 &	Physical activity and diet: n=22	Mobile app: n=22	Diet and adiposity-related outcomes
FloresMateo 2015	11	913	General population<br>Female & males<br>Range of mean ages: 22.2 to 44.9	Physical activity: n=5	Mobile app only: n=1<br>Mobile app & usual care: n=3<br>Mobile app & counselling: n=1<br>Mobile app & multimedia: n=5<br>Mobile app & program: n=2	Physical activity
Furness 2020	24	4583	Survivors of cancer<br>Female & males<br>Age: Adults aged 18+	Physical activity: n=9<br>Diet: n=13	Web-based: n=12<br>Telephone-based: n=9<br>Videoconferencing: n=1<br>Email: n=1<br>SMS & telehealth: n=1	Physical activity<br>Diet
Gal 2018	18	2734	General population & various chronic diseases<br>Female & males<br>Range of mean ages: 20.5 to 71.3	Physical activity: n=18	Mobile app: n=18	Physical activity
Gong 2023	15	2362	Survivors of cancer<br>Female & males	Diet: n=15	Website: n=4<br>Mobile app: n=1	Diet-related outcomes

Author, year, country	Studies (k=)	Total sample (n=)	Population or condition; Gender; Age mean±SD or range (years)	Target behaviours (n= studies)	Intervention details	Relevant outcomes of interest
			Range of mean ages: 40.03 to 73.05		Emails: n=1<br>Phone calls: n=6<br>Phone calls, website combination: n=1<br>Phone calls, Skype calls combination: n=1<br>text messages, emails, Facebook combination: n=1	
Jahangiry 2021	8	779	Adults with a BMI ≥ 25 kg/m2<br>Female & males<br>Range of mean ages: 41.4 to 57.6	Diet and physical activity: n=7	Web based n=8	Weight
Jahangiry 2017	22	30951	General population<br>Female & males<br>Range of mean ages: 19 to 64.9 years	Physical activity: n=22	Web based: n=22	Physical activity
Jung 2022	8	4728	General population<br>Female & males<br>Range of mean ages: 24.8 to 50.4	Physical activity: n=5<br>Sedentary behaviour: n=3	SMS text messages: n=2<br>Phone call, SMS & wearable activity monitor: n=1<br>Phone calls: n=3<br>App & wearable activity monitor: n=2	Physical activity
Kwan 2020	38	11194	Older adults, aged over 50 years<br>Female & males<br>Range of mean ages: 50.8 to 82	Physical activity: n=38	Automated advice: n=2<br>Tele-counselling: n=6<br>Digital-tailored advice: n=7<br>Digital physical activity recording: n=2<br>Digital physical activity coaching: n=18<br>Online resources: n=2),<br>Online social support: n=1<br>Physical activity auto-tracking feedback: n=15<br>Video demonstrations: n=3<br>Video games: n=1<br>Video vignettes: n=1<br>(Studies included multiple components)	Physical activity
Laranjo 2021	35	7454	General population<br>Female & males<br>Age rage: 18 to 65 years	Physical activity: n=35	Mobile app and activity tracker: n=12<br>Physical activity tracker: n=7<br>App alone: n=8<br>App or tracker: n=1	Physical activity
Lau 2020	15	5816	Adults with overweight or obesity<br>Female & males<br>Range of mean ages: 19.5 to 50.4	Physical activity & diet: n=15	Remote weight loss intervention: n=1<br>Computerised self-monitoring & technology-assisted feedback: n=1<br>Web-based weight loss program: n=1<br>Web-based weight management: n=2 Tailored dietary feedback & SMS support: n=1<br>Smartphone weight loss intervention: n=1<br>Workplace weight loss program: n=1<br>Web-based intervention: n=1<br>Web-based lifestyle app: n=1<br>E-learning to prevent weight gain: n=1<br>SMS for weight control: n=1	Weight

Author, year, country	Studies (k=)	Total sample (n=)	Population or condition; Gender; Age mean±SD or range (years)	Target behaviours (n= studies)	Intervention details	Relevant outcomes of interest
					Web-based behavioural weight loss program: n=1<br>Web-based behavioural counselling on weight loss: n=1<br>Computer automated tailored counselling: n=1	
Lau 2017	14	3169	Overweight (BMI ≥ 25 kg m^2) and/or obese (BMI ≥ 30 kg m^2) women during the perinatal period (starting from pregnancy to 1 year postpartum)<br>Females only<br>Range of mean ages: 24.2 to 33.9	Physical activity: n=8<br>Diet: n=6	Website & phone & device: n=1<br>SMS & software & in-person & booklet & device: n=1<br>Mobile apps & SMS & in-person & video & device: n=1<br>Website & email & in-person & software & dietary recalls: n=1<br>SMS & in-person & phone & magnets: n=1<br>SMS & in-person & postcards & device: n=1<br>Website & SMS & Facebook & phone & device: n=1<br>Website & SMS & Facebook & phone & device: n=1<br>Computer-based video & home-based & in-person & software & device/Community-based exercise & dietary intervention: n=1<br>Computer-based video & cueing sheet & worksheet: n=1<br>Automated postcards via internet & phone & in-person & device: n=1<br>SMS: n=1<br>Email & in-person & video & handbook & software & device: n=1<br>Website & forum & email & device & journal & calendar: n=1	Physical activity<br>Diet
Lee 2023	22	10486	Adults with insomnia<br>Females & males<br>Range of mean ages: 24.6 to 58.6	Sleep: n=22	Digital interventions (computer, web-based, & smartphone applications): not described	Sleep
Lundell 2015	9	982	Chronic obstructive pulmonary disease<br>Females & males<br>Range of mean ages: 64 to 73	Physical activity & self-management: n=9	Web-based: n=1<br>SMS: n=1<br>Website & mobile phone: n=2<br>Telephone & other: n=6<br><br>Live education using telehealthcare through Text chats n=1/9<br>Live education using telehealthcare through video-calls n=1/9	Physical activity
Lyzwinski 2014	12	1300	General population<br>Females & males<br>Range of mean ages: 20.4 to 57	Physical activity and diet: n=12	Mobile phone: n=8<br>Personal digital assistant: n=2<br>Podcast: n=2	Weight
Mancinelli 2022	7	1873	Pregnant women with sub-clinical symptoms<br>Women only<br>Range of mean ages: 28.9 to 33.8	Sleep: n=7	Web-based: n=5<br>Mobile app: n=2	Sleep

Author, year, country	Studies (k=)	Total sample (n=)	Population or condition; Gender; Age mean±SD or range (years)	Target behaviours (n= studies)	Intervention details	Relevant outcomes of interest
McMahon 2021	9	1329	Adult males with cardiovascular disease risk factors<br>Males only<br>Range of mean ages: 35.9 to 48.2	Physical activity & diet: n=8<br>Sedentary behaviour: n=1	Mobile app and website combination: n=1<br>Website only: n=8	Adiposity-related outcomes
Nour 2016	14	7984	Young adults (general populations)<br>Females & males<br>Range of mean ages: 18.1 to 27.7 years	Diet: n=14	Online education: n=4<br>Online education & emails: n=2<br>SMS & apps & email & website: n=2<br>Email: n=1<br>Phone calls: n=1<br>Website & email & phone calls: n=1<br>SMS & email: n=1<br>SMS: n=1<br>Online assessment & email: n=1	Diet
NúñezdeArenas-Arroyo 2021	18	4937	Healthy adults aged over 55 years<br>Females & males<br>Range of mean ages: 58 and 74.2	Physical activity: n=18	Mobile only: n=7<br>Website only: n=11	Physical activity
Oliveira 2022	16	6907	Adults 18-65 receiving conventional treatment for weight control<br>Females & males<br>Range of mean ages: 24 to 48	Physical activity and diet: n=16	Multimedia (Web-based, SMS & other): n=6<br>Emails: n=1<br>Email & SMS: n=1<br>App: n=1<br>Web conference: n=1<br>Website & medical care: n=2<br>Voice response & messages: n=1<br>Platform & SMS: n=1<br>Platform: n=1<br>Memory game: n=1	Adiposity-related outcomes
Park, 2019, Korea	20	2,318	Obese adults<br>Females & males<br>Mean age range: 22.6-63.1	Physical activity & diet: n=20	Standard tracking app: n=1<br>Text messaging, calls: n=8<br>Smartphone/app: n=6<br>Lifestyle weight loss coaching calls: n=1<br>Pedometer n=1<br>mHealth program: n=1<br>Podcast & mobile media: n=1<br>Smart scale intervention: n=1/20	Adiposity-related outcomes
Peng 2022A<br><br>*Asia Pac J Oncol Nurs*	9	1127	Breast cancer<br>Females only<br>Mean (range) age: 52 (47 to 62)	Physical activity: n=9	Web-based: n=3<br>Email: n=1<br>Wearable & telephone: n=1<br>Wearable & mobile app: n=1<br>Telephone: n=3	Physical activity and sedentary behaviour
Peng 2022B	22	8,333	College students<br>Female & male	Physical activity & sedentary behaviour: n=22	Smartphone: n=8<br>Social media: n=4	Physical activity

Author, year, country	Studies (k=)	Total sample (n=)	Population or condition; Gender; Age mean±SD or range (years)	Target behaviours (n= studies)	Intervention details	Relevant outcomes of interest
			Mean age range: 16 to 27.8		Wearables: n=5<br>Online/web-based: n=5	
Rhodes 2020	11	3,280	Pregnant Women<br>Female only<br>Adults aged 18+	Physical activity & diet: n=5<br>Physical activity: n=4<br>Diet: n=2	Text messages: n=4<br>Web-based/website: n=4<br>Mobile app: n=3	Weight
Rodríguez-Torres 2023	10	1,835	Thoracic malignancies<br>Females & males<br>Mean age range: 44.6 to 59.18	Physical activity: n=9<br>Physical activity & diet: n=1	Website: n=2<br>Mobile app: n=3<br>Email & website: n=1<br>Phone: n=1<br>Email: n=1<br>Online sheet: n=1<br>Fitbit app & phone: n=1	Physical activity
Romeo 2019	9	1,740	General population, including with a health condition<br>Females & males<br>Mean age range: 33.7 to 63.0	Physical activity: n= 9	Mobile apps: n=9	Physical activity
Seyffert, 2016, United States	15	2,392	Insomnia<br>Females & males<br>Mean age range: 37-53	Sleep: n=15	Internet/web-based: n=15	Sleep
Shi, 2023, Japan	91	7,062	Overweight or Obese<br>Female & male<br>Mean age range: 22.7-69.0	Physical activity & diet: n=51	Smartphone n=25<br>Website (computer) & smartphone n=1<br>Website (computer) n=71	Weight
Smith, 2020, United States	59	8,742	Overweight/inactive, with chronic conditions<br>Female & male<br>Mean age: 42.2	Physical activity: n=59	Text messages: n=11<br>Text message & additional support (e.g., phone calls): n=16<br>Smartphone: n=4<br>Text message & in person counselling: n=9<br>Text message & website: n=4<br>Text message & in person support n=6	Physical activity
Stavric, 2022, Canada	16	2,439	Chronic health conditions<br>Female & male<br>Mean age range: 42.4-67.4	Physical activity: n=16	Website: n=10<br>Specifically designed platforms: n=3<br>Apps: n=3	Physical activity
Wang 2023	16	799	Post-stroke<br>Female & male<br>Adults aged 18 &	Physical activity & sedentary behaviour: n=11<br>Other: n=5	Phone/tablet: n=3<br>Virtual reality: n=5<br>Video games: n=4<br>Monitoring devices: n=4	Physical activity<br>Sedentary behaviour
Xu, 2021, China	16	3,898	Coronary heart disease<br>Female & male<br>Mean age range: 38-78	Physical activity only: n=3<br>Diet only: n=2<br>Diet & physical activity: n=11	Smartphone app n=14<br>SMS: n=1<br>Telephone calls n=1	Diet and adiposity-related outcomes
Yerrakalva 2019, UK	5	486	Community-dwelling older adults<br>Female & males<br>55 years & older	Physical activity: n=3<br>Sedentary behaviour: n=2	Mhealth app n=5	Physical activity
Yu, 2023, China	18	3,261	Cardiac rehabilitation patients<br>Females & males	Physical activity: n=18	Websites: n=12*<br>Smartphone apps: n=9*	Physical activity Moderate-intensity physical activity

Author, year, country	Studies (k=)	Total sample (n=)	Population or condition; Gender; Age mean±SD or range (years)	Target behaviours (n= studies)	Intervention details	Relevant outcomes of interest
			Mean age range: 54-71		Telehealth: n=8*<br>Wearable trackers: n=14*<br>*Interventions involves multiple components	Physical activity
Zhang, 2022, China	8	1,495	Physically inactive adults<br>Female & male<br>65 years & older	Physical activity only: n=4<br>Physical activity & diet: n=2<br>Physical activity & sedentary behaviour: n=2	Mobile app only: n=2<br>Mobile app & other intervention strategies (multicomponent interventions): n=6	Physical activity<br>Sedentary behaviour

Supplementary Table 2. AMSTAR 2 quality appraisal of reviews.

Review	AMSTAR 2 Items																Overall confidence rating
Item number	1	2	3	4	5	6	7	8	9	10	11	12	13	14	15	16	
Alhussein 2022	N	PY	N	Y	Y	Y	N	PY	Y	N	Y	Y	Y	Y	Y	Y	Low
Alsahli 2022	Y	Y	Y	PY	Y	Y	Y	Y	Y	N	Y	N	Y	Y	N	Y	Low
Arambepola 2016	Y	Y	Y	PY	Y	Y	N	PY	Y	N	Y	N	N	Y	Y	Y	Critically low
Barnett 2022	Y	Y	Y	PY	Y	N	Y	Y	Y	N	Y	Y	Y	Y	N	Y	Low
Beleigoli 2019	N	N	N	PY	Y	N	N	Y	Y	N	Y	N	Y	Y	Y	Y	Critically low
Berry 2021	Y	Y	Y	PY	Y	N	N	Y	Y	N	Y	N	Y	Y	Y	Y	Low
Bunting 2021	Y	PY	N	PY	Y	Y	Y	Y	Y	N	Y	N	Y	N	N	Y	Low
Chew 2022	Y	Y	Y	PY	Y	Y	N	Y	Y	Y	Y	Y	Y	Y	Y	Y	Low
David 2022	Y	Y	Y	PY	Y	Y	Y	Y	Y	Y	Y	Y	Y	Y	Y	Y	High
Deng 2023	Y	Y	Y	Y	Y	Y	Y	PY	Y	N	Y	N	N	Y	Y	Y	Low
Duan 2021	Y	PY	N	PY	N	Y	Y	PY	Y	N	Y	N	Y	Y	Y	Y	Moderate
Emmerson 2019	Y	PY	Y	PY	Y	Y	N	PY	Y	N	Y	N	N	Y	N	N	Critically low
FakihElKhoury 2019	Y	Y	Y	PY	Y	Y	Y	Y	Y	Y	Y	Y	Y	Y	Y	Y	High
FloresMateo 2015	Y	N	N	PY	Y	Y	N	PY	Y	N	Y	Y	Y	Y	Y	Y	Critically low
Furness 2020	Y	PY	Y	PY	Y	N	Y	PY	Y	Y	Y	Y	N	Y	N	Y	Critically low
Gal 2018	Y	Y	Y	PY	Y	Y	N	PY	Y	N	Y	Y	Y	Y	Y	Y	Low
Gong 2023	Y	N	N	N	Y	N	N	PY	Y	Y	Y	N	N	Y	Y	Y	Critically low
Jahangiry 2021	Y	Y	N	N	Y	Y	N	N	Y	N	Y	Y	N	N	Y	Y	Critically low
Jahangiry 2017	Y	Y	N	N	Y	N	N	N	Y	N	Y	Y	Y	Y	Y	Y	Critically low
Jung 2022	Y	N	Y	N	Y	Y	N	Y	Y	N	Y	Y	Y	Y	Y	Y	Critically low
Kwan 2020	Y	N	N	PY	Y	Y	N	Y	Y	N	Y	Y	Y	Y	N	Y	Critically low
Laranjo 2021	Y	Y	N	PY	Y	Y	Y	PY	Y	Y	Y	Y	N	Y	Y	Y	Low
Lau 2020	Y	Y	Y	PY	Y	Y	N	Y	Y	Y	Y	Y	Y	Y	Y	Y	Low
Lau 2017	Y	Y	Y	PY	Y	Y	Y	Y	Y	N	Y	Y	Y	Y	Y	Y	High
Lee 2023	Y	Y	Y	N	Y	Y	N	PY	Y	N	Y	Y	Y	Y	Y	Y	Critically low
Lundell 2015	Y	Y	Y	N	Y	Y	N	PY	Y	Y	Y	N	Y	Y	Y	Y	Critically low
Lyzwinski 2014	Y	N	Y	PY	N	N	N	PY	Y	N	Y	N	N	Y	Y	Y	Critically low
Mancinelli 2022	Y	Y	Y	PY	Y	Y	N	Y	Y	N	Y	N	Y	N	Y	Y	Low
McMahon 2021	Y	Y	Y	PY	Y	Y	Y	Y	Y	Y	Y	N	Y	Y	Y	Y	High
Nour 2016	Y	Y	Y	N	Y	Y	Y	Y	Y	N	Y	N	N	N	Y	Y	Critically low

Review	AMSTAR 2 Items																Overall confidence rating
Item number	1	2	3	4	5	6	7	8	9	10	11	12	13	14	15	16	
NúñezdeArenas-Arroyo 2021	Y	Y	Y	PY	Y	Y	N	Y	Y	N	Y	Y	Y	Y	Y	Y	Low
Oliveira 2022	Y	Y	Y	PY	Y	N	N	PY	Y	N	Y	Y	N	N	N	Y	Critically low
Park 2019	Y	N	Y	N	N	Y	N	PY	Y	Y	Y	N	N	N	N	Y	Critically low
Peng 2022	Y	Y	Y	N	Y	Y	Y	Y	Y	Y	Y	Y	Y	Y	Y	Y	Low
Peng 2022	Y	Y	Y	PY	Y	Y	N	PY	Y	N	Y	N	N	N	N	Y	Critically low
Rhodes 2020	Y	Y	Y	PY	Y	Y	N	PY	Y	N	Y	N	Y	Y	N	Y	Critically low
Rodríguez-Torres 2023	Y	Y	Y	N	Y	Y	N	PY	Y	N	Y	Y	Y	Y	N	Y	Critically low
Romeo 2019	Y	N	Y	PY	Y	Y	N	Y	Y	N	Y	Y	N	Y	N	Y	Critically low
Seyffert 2016	Y	Y	Y	PY	Y	Y	N	PY	Y	Y	Y	N	Y	Y	Y	Y	Low
Shi 2023	Y	Y	Y	PY	Y	Y	N	N	Y	N	Y	N	Y	Y	N	Y	Critically low
Smith 2020	Y	Y	Y	N	Y	Y	N	Y	Y	Y	Y	Y	Y	Y	Y	Y	Critically low
Stavric 2022	Y	Y	Y	PY	Y	N	N	PY	Y	N	Y	N	Y	N	N	Y	Critically low
Wang 2023	Y	Y	Y	N	Y	N	N	N	Y	N	Y	N	N	Y	Y	Y	Critically low
Xu 2021	Y	N	Y	PY	Y	Y	N	PY	Y	N	Y	N	Y	Y	Y	Y	Critically low
Yerrakalva 2019	Y	Y	Y	PY	Y	Y	N	PY	Y	N	Y	N	N	N	N	Y	Critically low
Yu 2023	Y	Y	Y	PY	Y	Y	N	PY	Y	N	Y	Y	Y	Y	Y	Y	Low
Zhang 2022	Y	N	Y	PY	Y	Y	N	PY	Y	N	Y	N	N	N	N	Y	Critically low

Note: Y=yes; N=no; Partial Y=meets criteria for partial yes.

Legend: AMSTAR 2 Items: 1) The Participant, Intervention, Comparator and Outcome (PICO) components included in the review research question and inclusion criteria; 2) Explicit statement included that review methods were established prior to conduct and significant deviations justified; 3) Selection of included study designs explained; 4) Comprehensive search strategy used; 5) Study selection performed in duplicate; 6) Data extraction performed in duplicate; 7) List of excluded studies with justification provided; 8) Included studies described in adequate detail; 9) Satisfactory technique used for assessing risk of bias in included studies; 10) Sources of funding for included studies reported; 11) Appropriate methods for statistical combination of results used if meta-analysis performed; 12) Potential impact of risk of bias of individual studies assessed if meta-analysis performed; 13) Risk of bias of individual studies accounted for in discussion of the review results; 14) Any heterogeneity observed in the review results was explained and discussed; 15) Publication bias investigated and discussed if meta-analysis performed; 16) Authors reported any potential sources of conflict of interest.

Overall ratings

High: No or one non-critical weakness: the systematic review provides an accurate and comprehensive summary of the results of the available studies that address the question of interest.

Moderate: More than one non-critical weakness*: the systematic review has more than one weakness but no critical flaws. It may provide an accurate summary of the results of the available studies that were included in the review.

Low: One critical flaw with or without non-critical weaknesses: the review has a critical flaw and may not provide an accurate and comprehensive summary of the available studies that address the question of interest.

Critically low: More than one critical flaw with or without non-critical weaknesses: the review has more than one critical flaw and should not be relied on to provide an accurate and comprehensive summary of the available studies.

Supplementary Figure 1. Funnel plot for total physical activity (standardised mean difference)

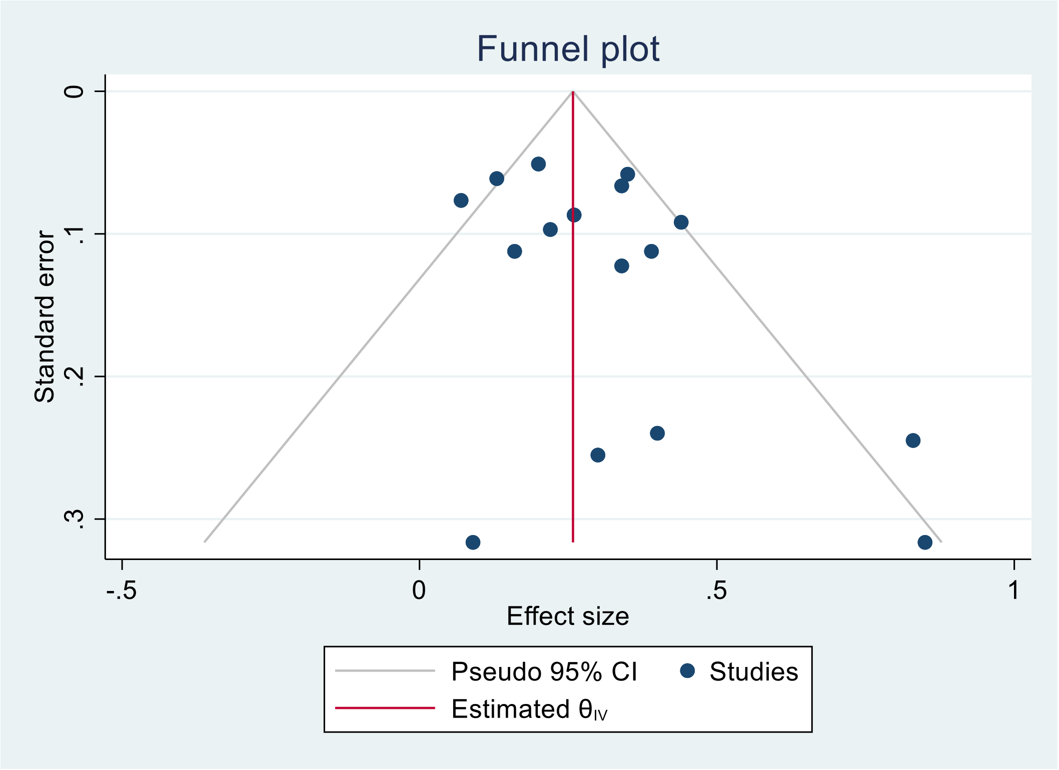

Supplementary Figure 2. Funnell plot for weight (mean difference)

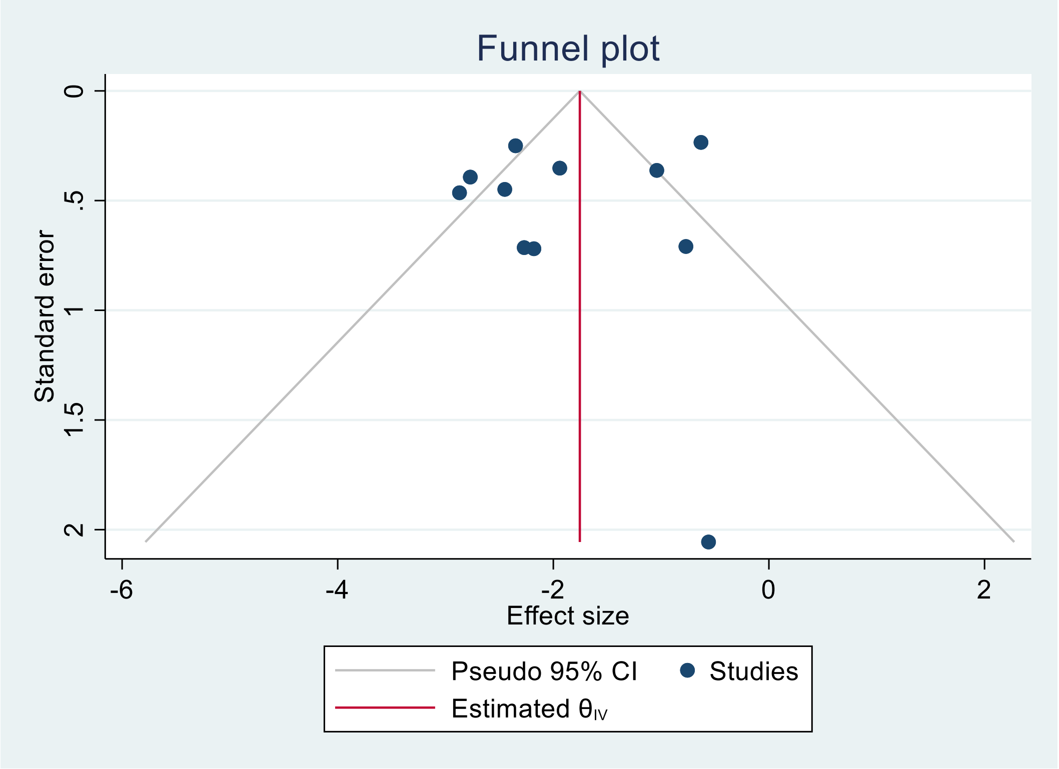

Supplementary Figure 3. Meta-analyses of the effects of E- and M-health interventions on total physical activity at follow-up (mean differences; mins/week)

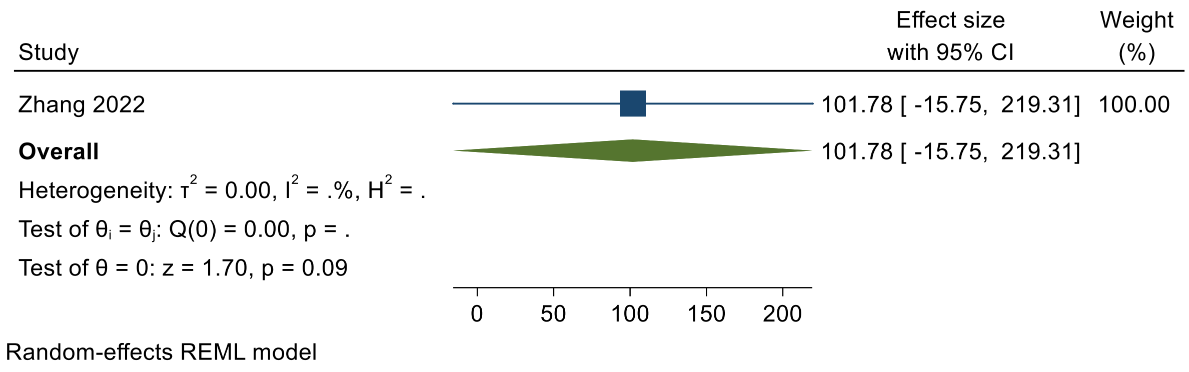

Supplementary Figure 4. Meta-analysis of the effects of E- and M-health interventions on total physical activity outcomes, standardised mean differences.

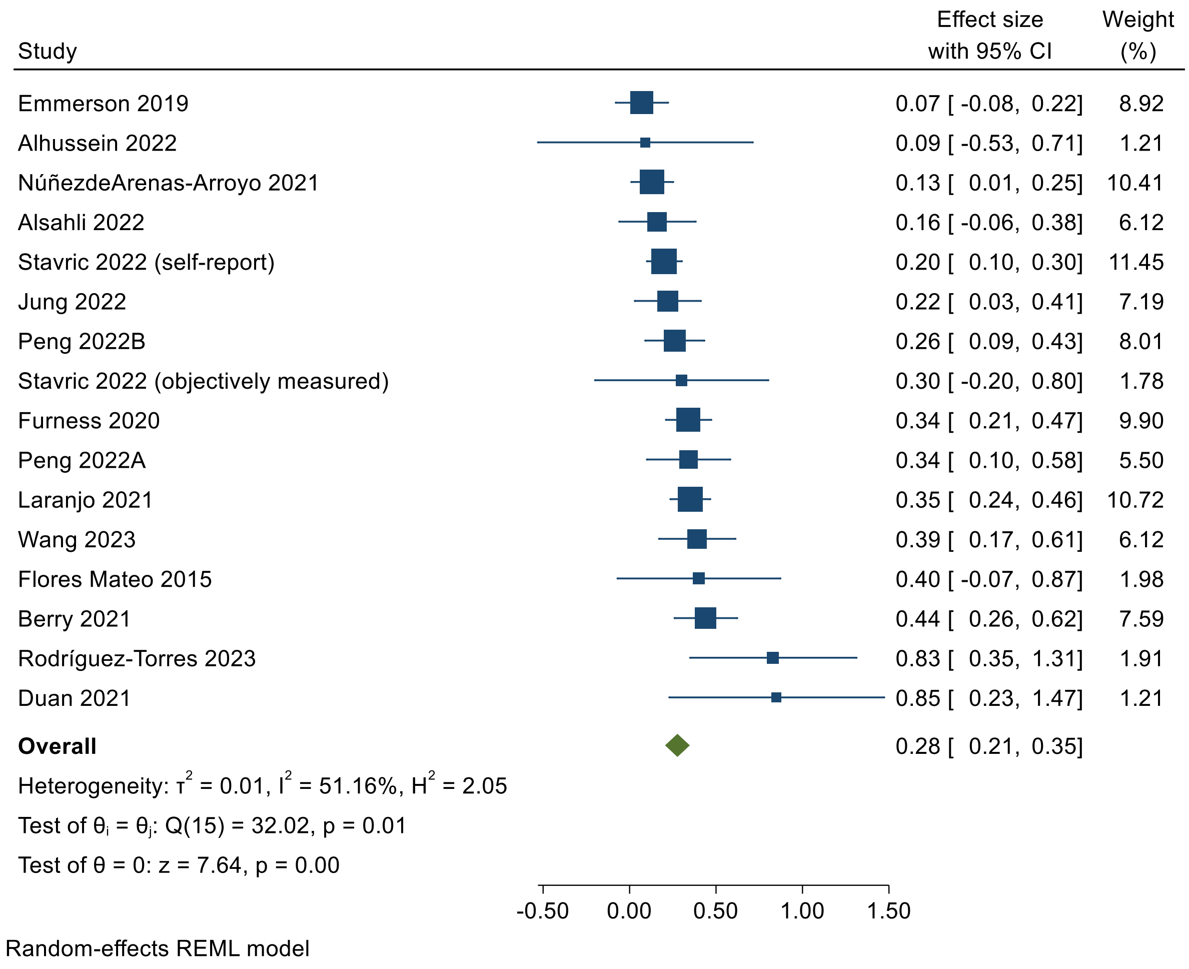

Supplementary Figure 5. Meta-analyses of the effects of E- and M-health interventions on total physical activity at follow-up, standardised mean differences.

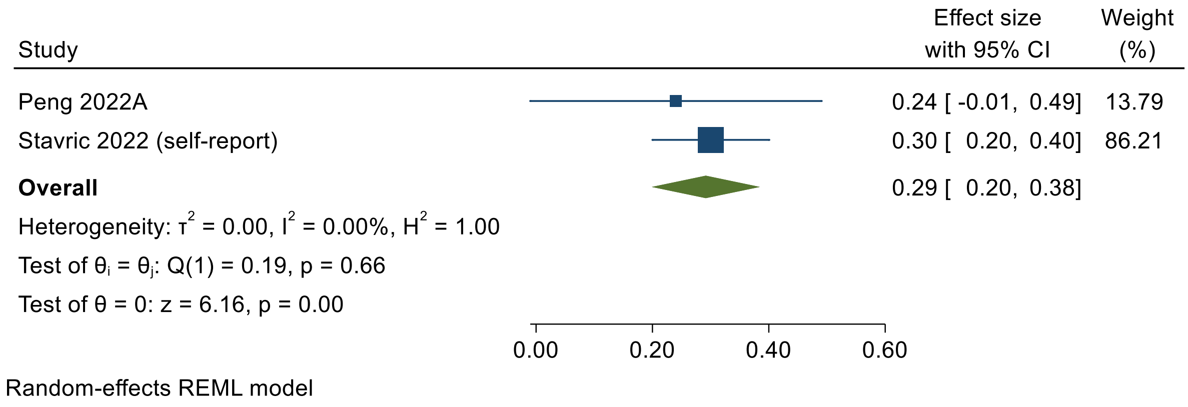

Supplementary Figure 6. Meta-analyses of the effects of E- and M-health interventions on moderate-to-vigorous physical activity at follow-up (mean differences; mins/week).

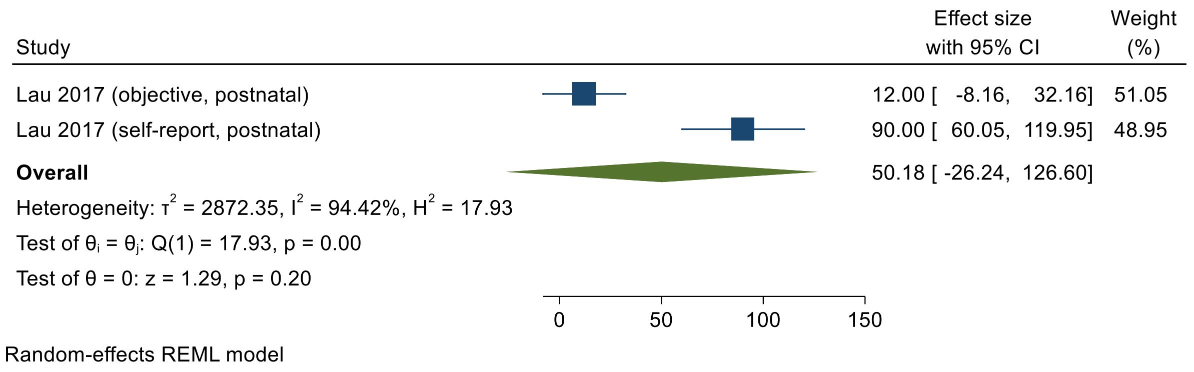

Supplementary Figure 7. Meta-analyses of the effects of E- and M-health interventions on moderate-to-vigorous physical activity, standardised mean differences.

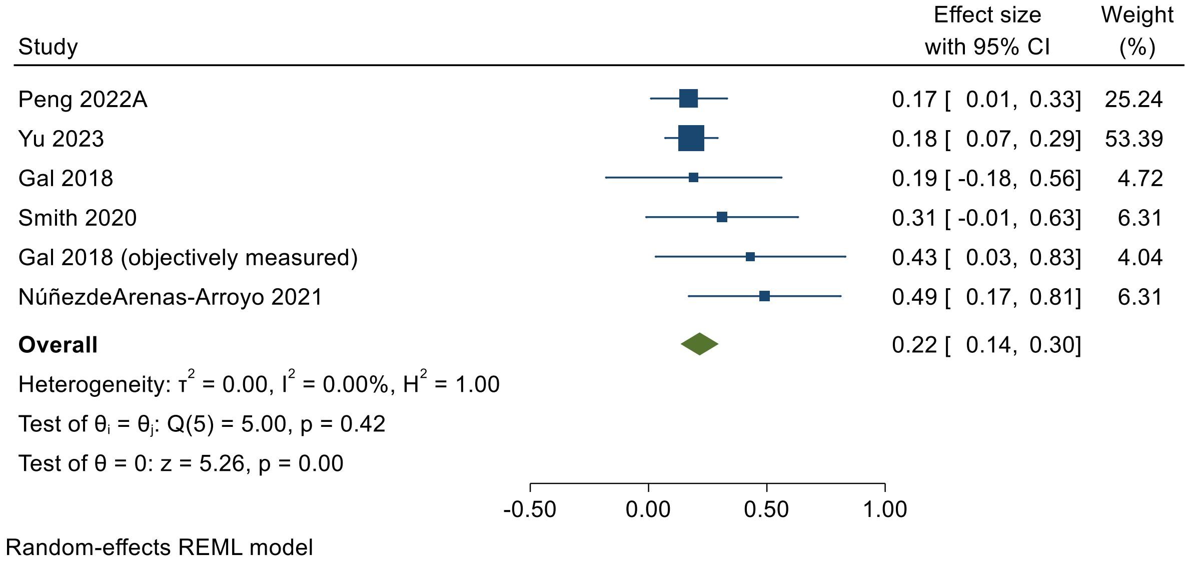

Supplementary Figure 8. Meta-analyses of the effects of E- and M-health interventions on steps per day at follow-up (mean differences; steps/day).

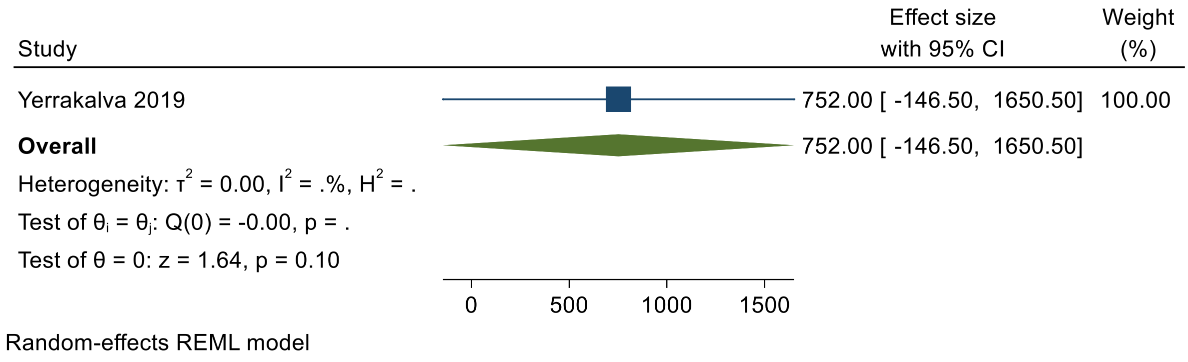

Supplementary Figure 9. Meta-analyses of the effects of E- and M-health interventions on daily steps, standardised mean differences.

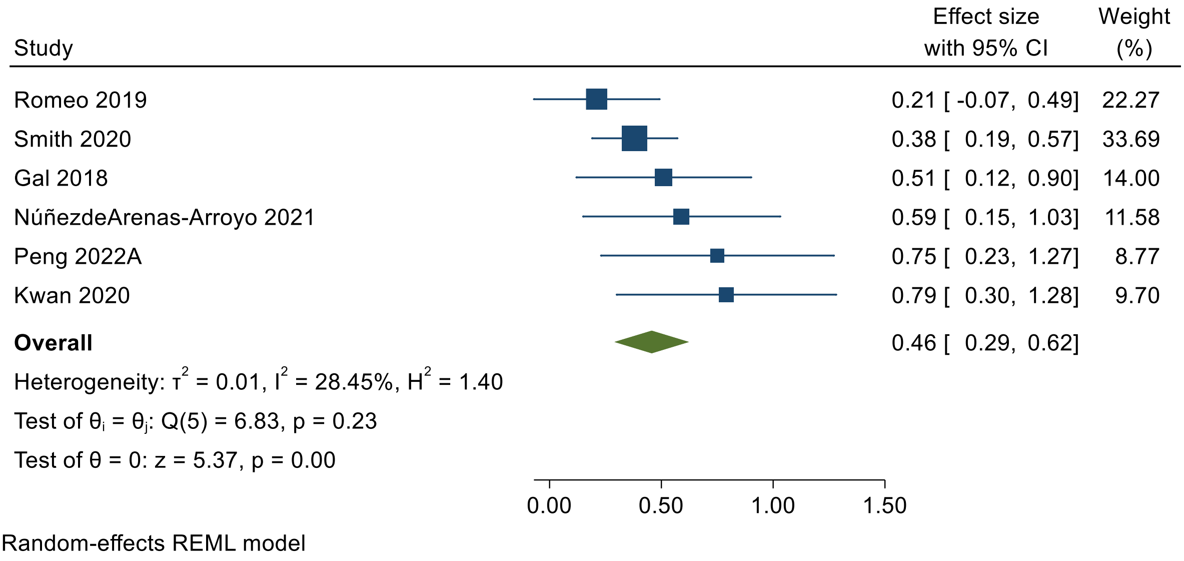

Supplementary Figure 10. Meta-analyses of the effects of E- and M-health interventions on fruit and vegetable consumption (mean differences; servings/day).

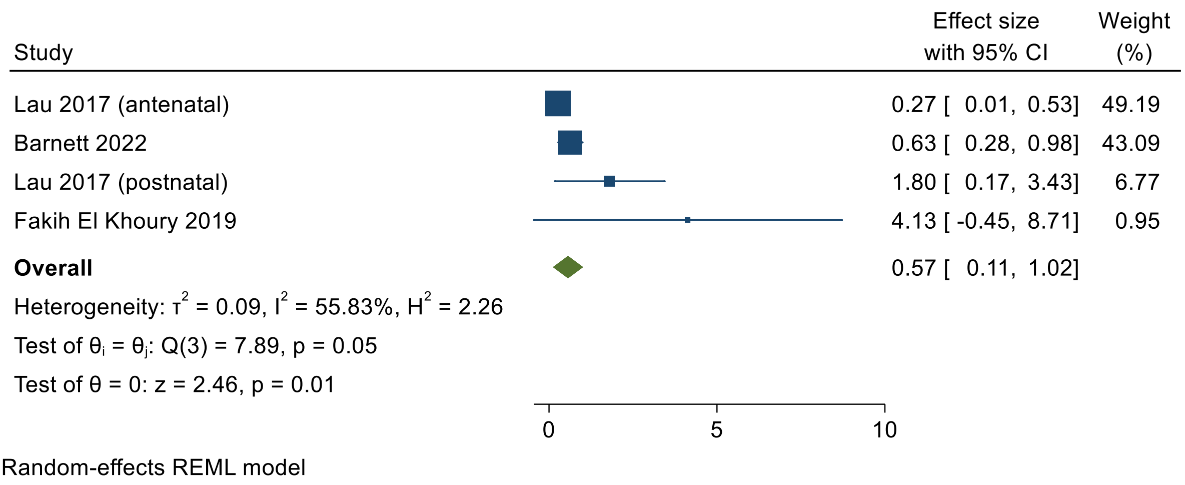

Supplementary Figure 11. Meta-analysis of the effects of E- and M-health interventions on fruit and vegetable consumption (standardised mean differences).

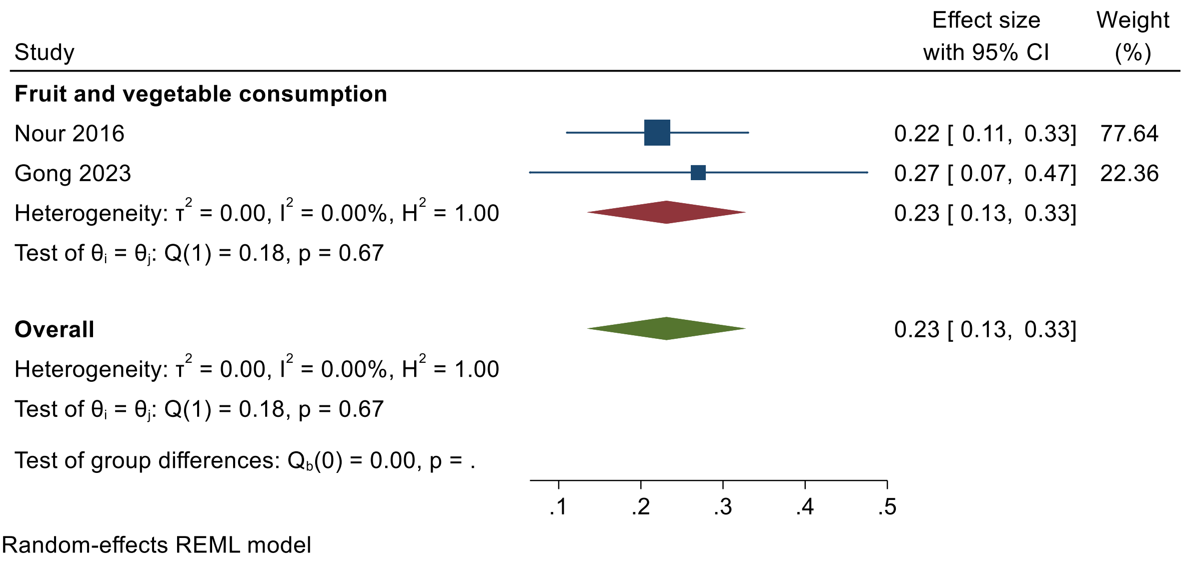

Supplementary Figure 12. Meta-analyses of the effects of E- and M-health interventions on saturated fat consumption (mean differences; grams/day).

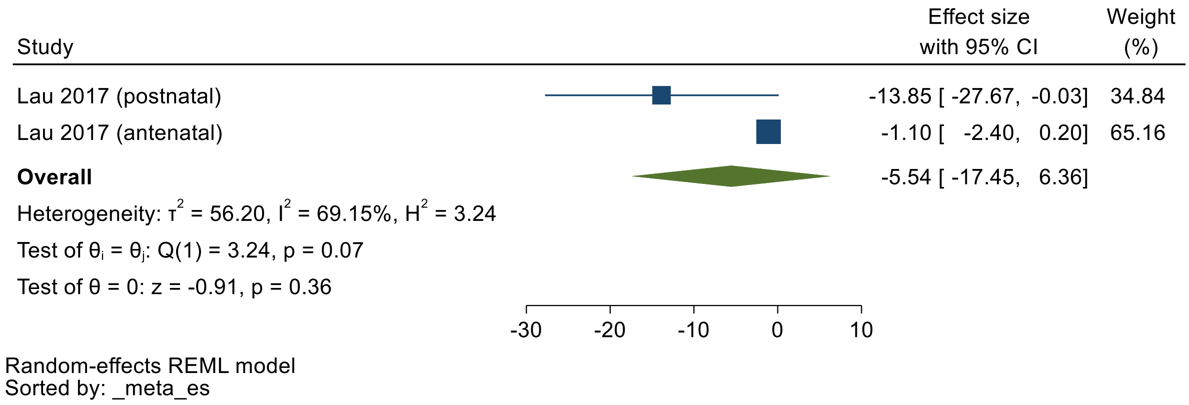

*Negative values = improvement

Supplementary Figure 13. Meta-analysis of the effects of E- and M-health interventions on sleep quality (standardised mean differences).

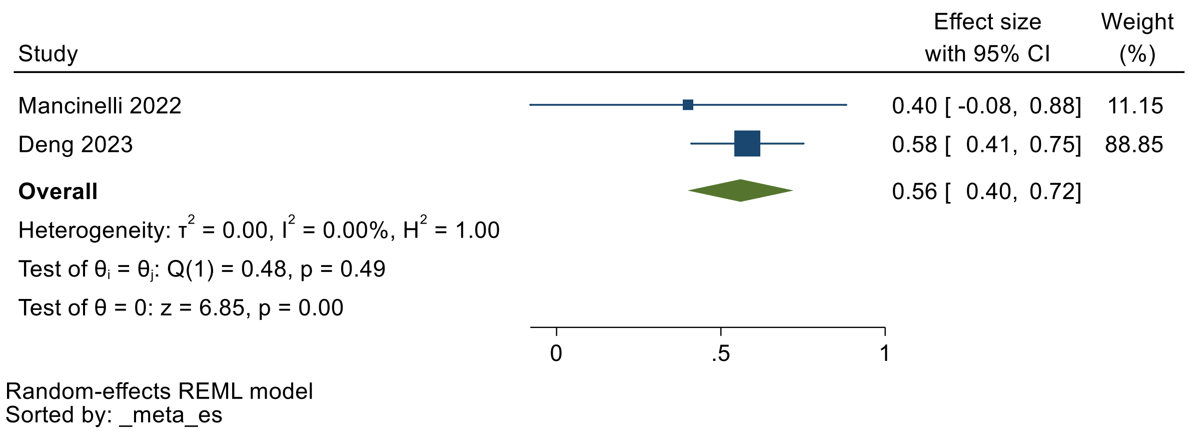

Supplementary Figure 14. Meta-analysis of the effects of E- and M-health interventions on insomnia severity (standardised mean differences).

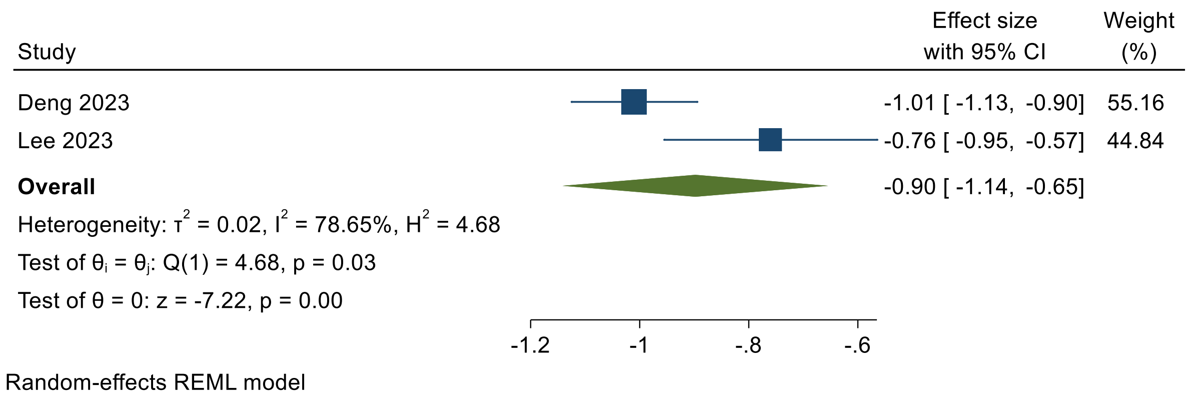

*Negative values = improvement

Supplementary Figure 15. Meta-analyses of the effects of E- and M-health interventions on weight change (mean differences; kgs).

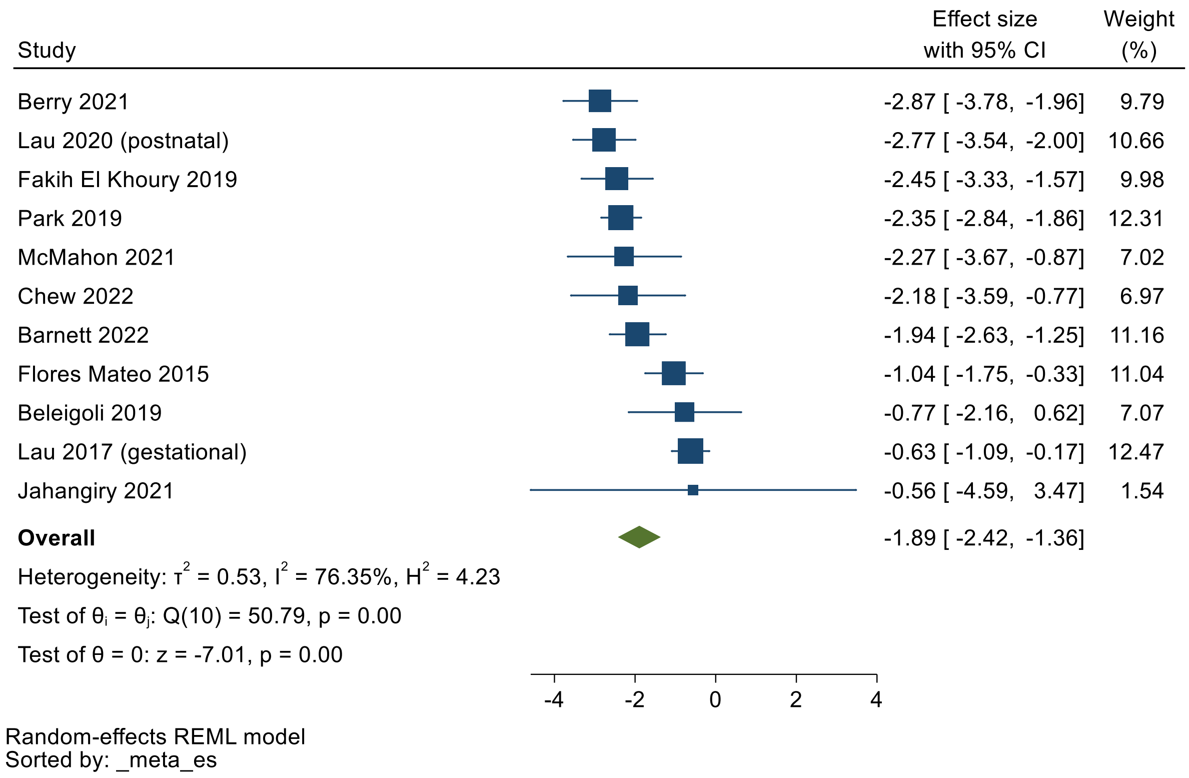

Supplementary Figure 16. Meta-analysis of the effects of E- and M-health interventions on weight change (standardised mean differences).

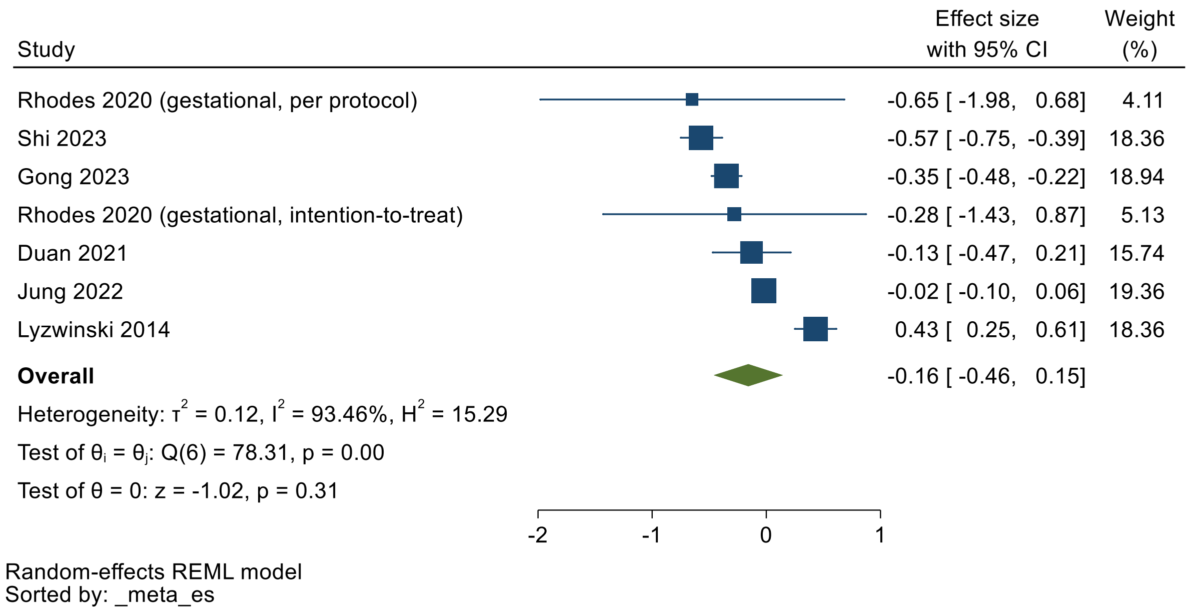

*Negative values = improvement

Supplementary Figure 17. Subgroup analyses for total PA (mean difference, min/week).

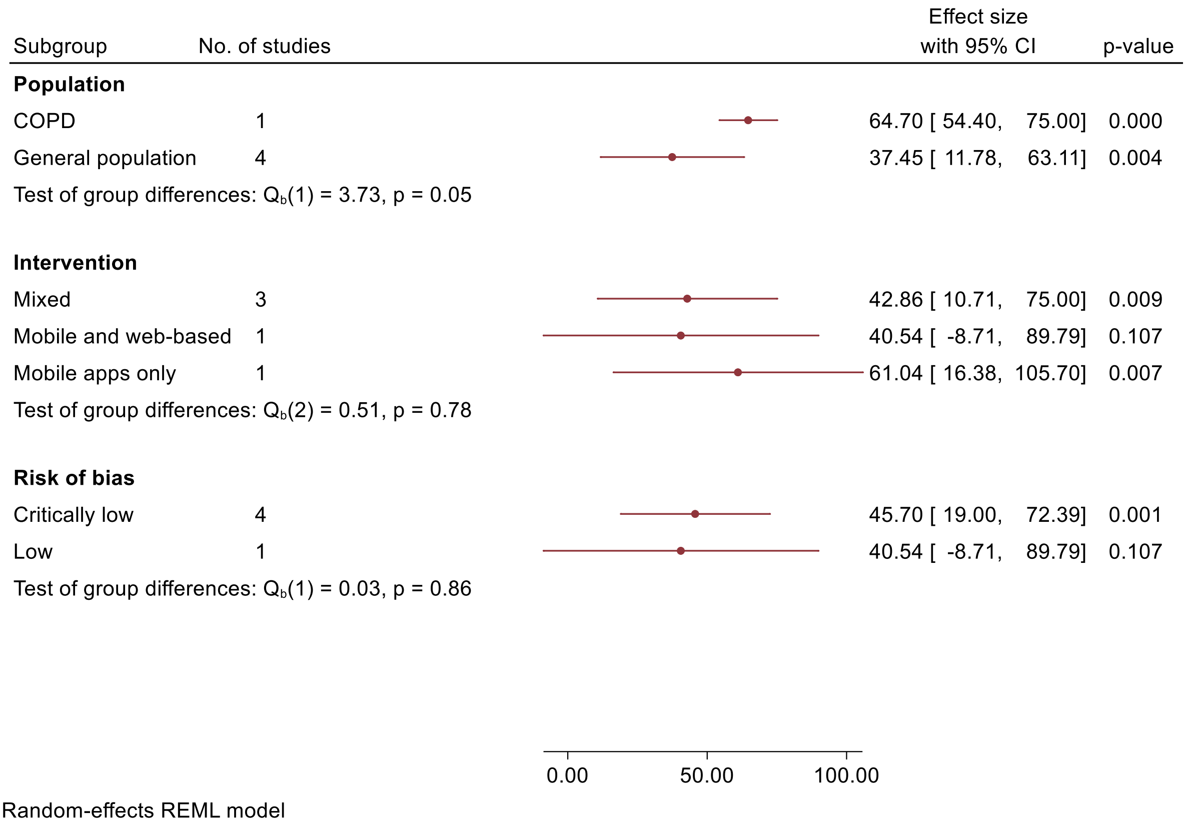

NOTE: The risk of bias assessment was performed using the AMSTAR-2 tool, with ratings categorised as "high ", "moderate", "low", or "critically low" confidence.

Supplementary Figure 18. Subgroup analyses for total PA (standardised mean difference).

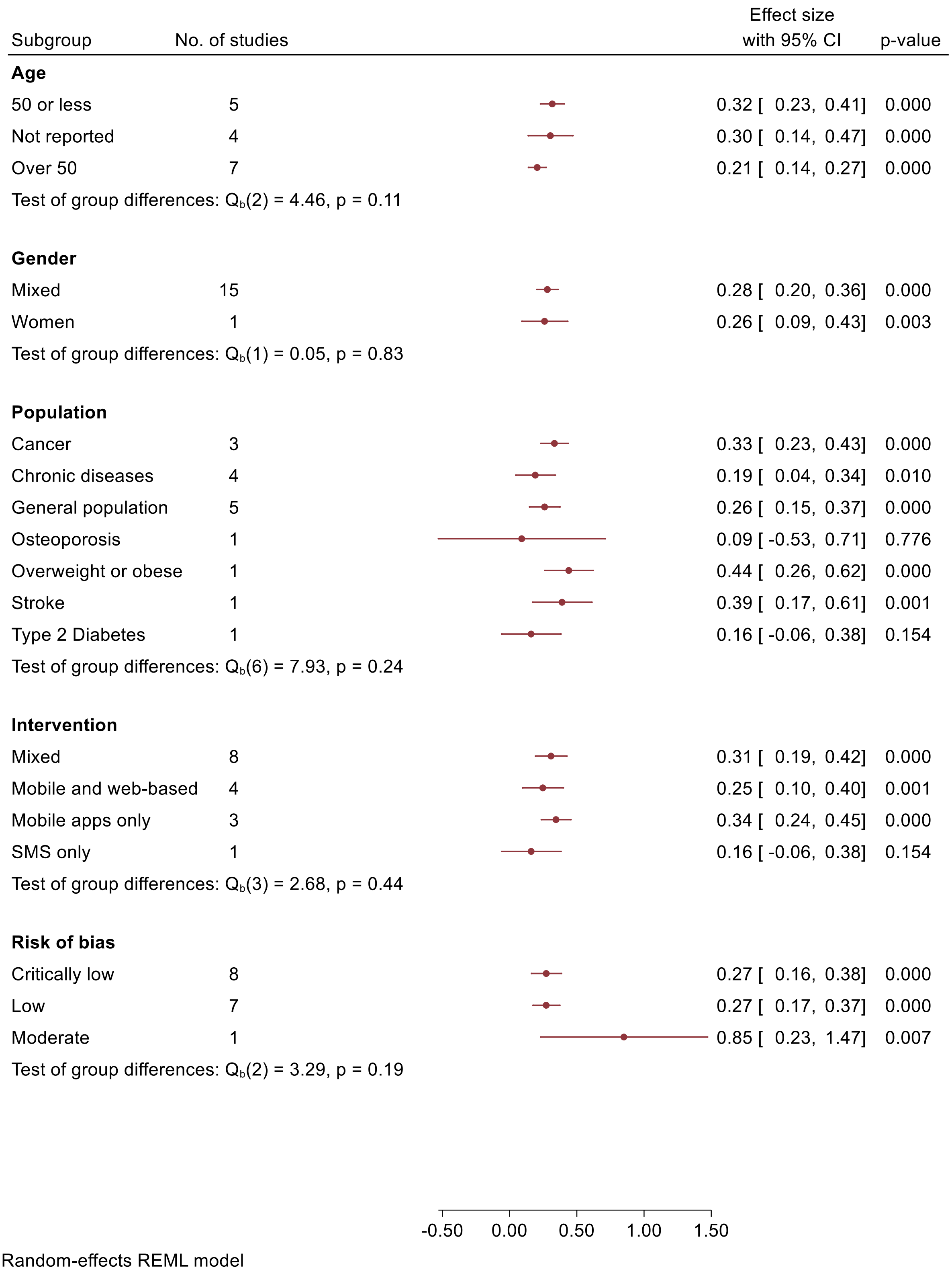

NOTE: The risk of bias assessment was performed using the AMSTAR-2 tool, with ratings categorised as "high ", "moderate", "low", or "critically low" confidence.

Supplementary Figure 19. Subgroup analyses for MVPA (mean difference, min/week)

Subgroup	No. of studies	Effect size with 95% CI	p-value
**Population**			
General population	2	30.29 [-10.80, 71.39]	0.149
Overweight or obese	4	74.82 [10.46, 139.19]	0.023
Test of group differences: $Q_b(1) = 1.31$, $p = 0.25$			
**Intervention**			
Mixed	4	74.82 [10.46, 139.19]	0.023
Mobile and web-based	1	56.35 [17.43, 95.27]	0.005
Web-based only	1	13.42 [12.96, 13.88]	0.000
Test of group differences: $Q_b(2) = 8.17$, $p = 0.02$			
**Risk of bias**			
Critically low	1	13.42 [12.96, 13.88]	0.000
High	4	74.82 [10.46, 139.19]	0.023
Low	1	56.35 [17.43, 95.27]	0.005
Test of group differences: $Q_b(2) = 8.17$, $p = 0.02$			

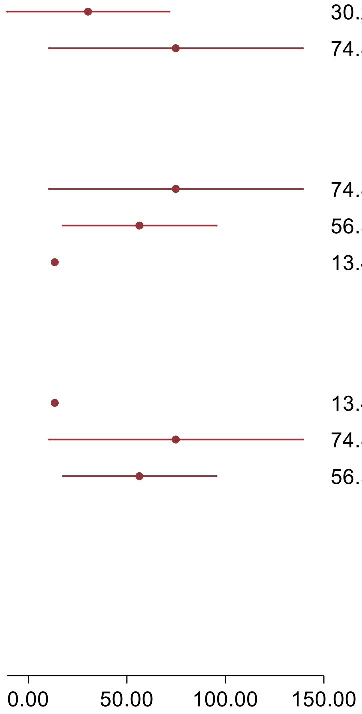

Random-effects REML model

NOTE: The risk of bias assessment was performed using the AMSTAR-2 tool, with ratings categorised as "high ", "moderate", "low", or "critically low" confidence.

Supplementary Figure 20. Subgroup analyses for MVPA (standardised mean difference).

Sugbroup	No. of studies	Effect size with 95% CI	p-value
**Age**			
50 or less	4	0.22 [0.09, 0.35]	0.001
Over 50	2	0.30 [0.00, 0.59]	0.048
Test of group differences: $Q_b(1) = 0.22$, $p = 0.64$			
**Population**			
Cardiometabolic diseases	1	0.18 [0.07, 0.29]	0.001
General population	4	0.28 [0.11, 0.45]	0.002
Overweight or obese	1	0.31 [-0.01, 0.63]	0.058
Test of group differences: $Q_b(2) = 1.28$, $p = 0.53$			
**Intervention**			
Mixed	2	0.18 [0.09, 0.27]	0.000
Mobile and web-based	1	0.49 [0.17, 0.81]	0.003
Mobile apps only	2	0.30 [0.03, 0.57]	0.030
SMS only	1	0.31 [-0.01, 0.63]	0.058
Test of group differences: $Q_b(3) = 4.24$, $p = 0.24$			
**Risk of bias**			
Critically low	1	0.31 [-0.01, 0.63]	0.058
Low	5	0.21 [0.13, 0.29]	0.000
Test of group differences: $Q_b(1) = 0.36$, $p = 0.55$			

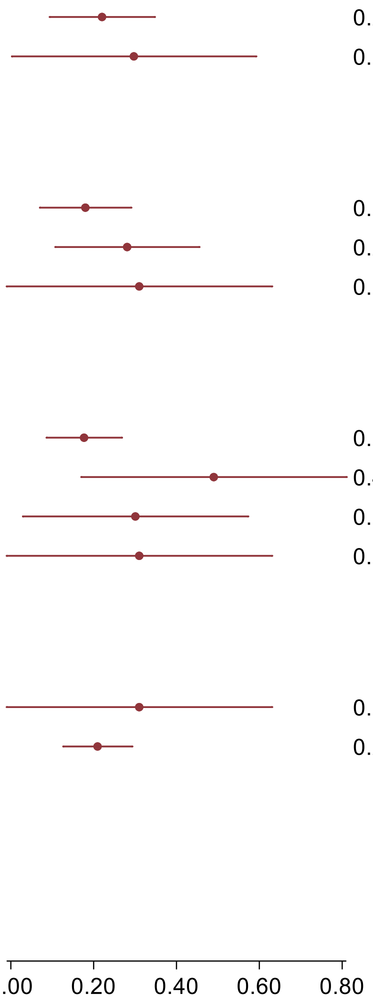

Random-effects REML model

NOTE: The risk of bias assessment was performed using the AMSTAR-2 tool, with ratings categorised as "high ", "moderate", "low", or "critically low" confidence.

Supplementary Figure 21. Subgroup analyses for steps (mean difference, steps/day).

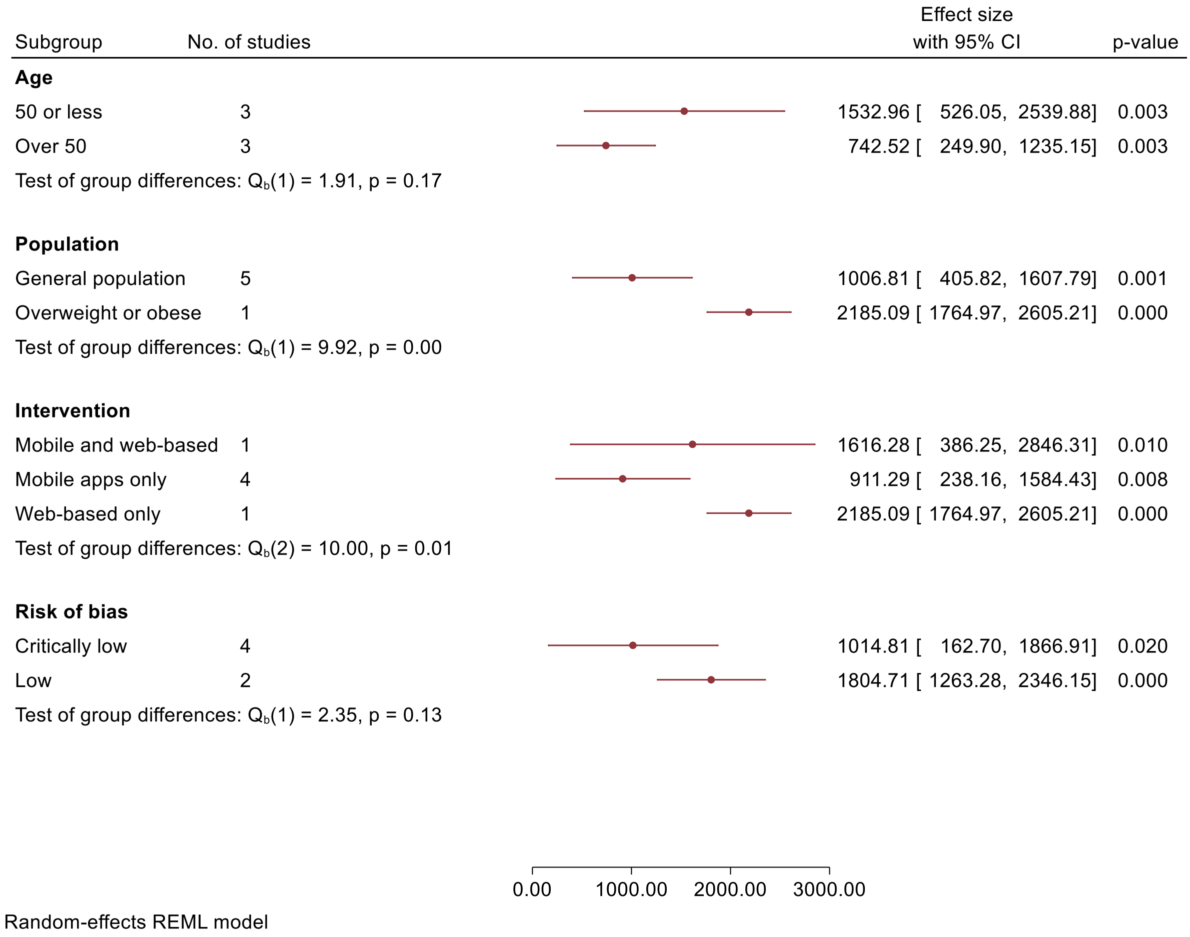

NOTE: The risk of bias assessment was performed using the AMSTAR-2 tool, with ratings categorised as "high ", "moderate", "low", or "critically low" confidence.

Supplementary Figure 22. Subgroup analyses for steps (standardised mean differences).

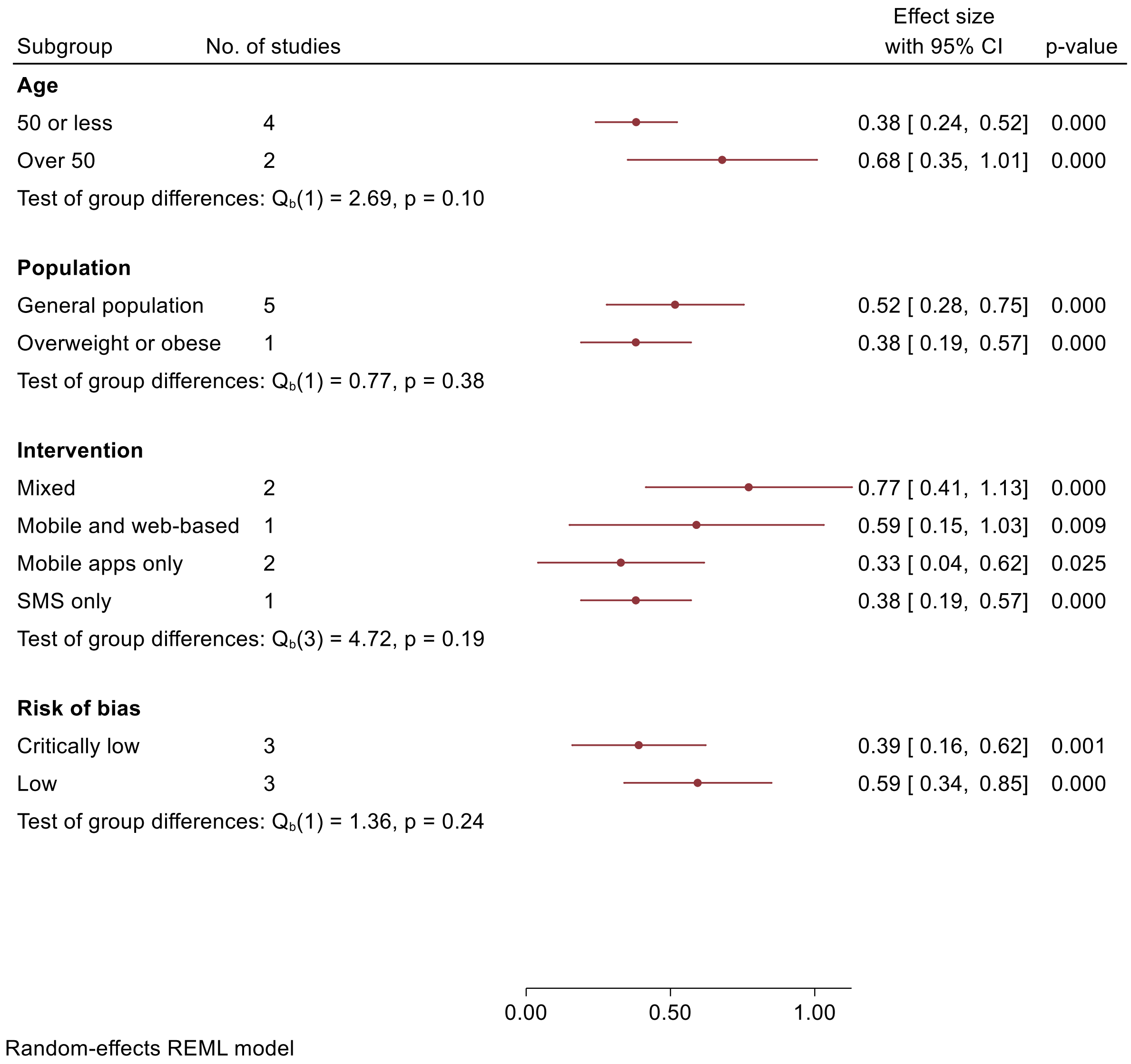

NOTE: The risk of bias assessment was performed using the AMSTAR-2 tool, with ratings categorised as "high ", "moderate", "low", or "critically low" confidence.

Supplementary Figure 23. Subgroup analyses for on fruit and vegetable consumption (mean difference, servings/day)

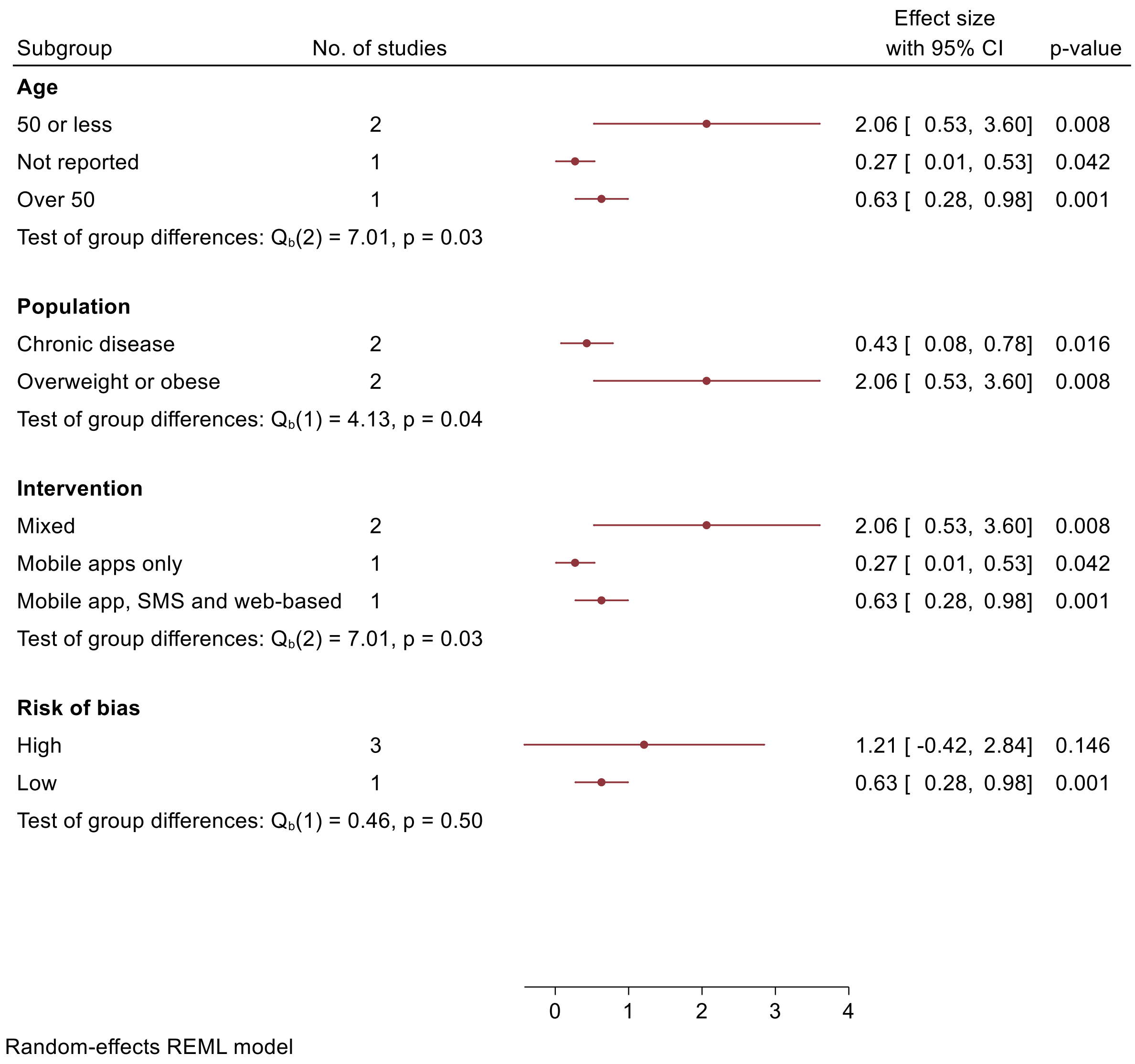

NOTE: The risk of bias assessment was performed using the AMSTAR-2 tool, with ratings categorised as "high ", "moderate", "low", or "critically low" confidence.

Supplementary Figure 24. Subgroup analyses for energy intake (mean differences, kcals/day).

Subgroup	No. of studies	Effect size with 95% CI	p-value
**Age**			
50 or less	5	-52.77 [-109.34, 3.79]	0.067
Not reported	2	-156.76 [-215.09, -98.42]	0.000
Test of group differences: $Q_b(1) = 6.29$, $p = 0.01$			
**Population**			
Chronic disease	1	-149.52 [-215.78, -83.26]	0.000
Overweight or obese	6	-84.32 [-154.05, -14.59]	0.018
Test of group differences: $Q_b(1) = 1.77$, $p = 0.18$			
**Intervention**			
Mixed	4	-52.12 [-109.24, 5.00]	0.074
Mobile and web-based	1	-181.70 [-304.72, -58.68]	0.004
Mobile apps only	2	-147.90 [-213.30, -82.49]	0.000
Test of group differences: $Q_b(2) = 6.43$, $p = 0.04$			
**Risk of bias**			
High	5	-88.56 [-156.40, -20.72]	0.011
Low	2	-173.75 [-291.54, -55.96]	0.004
Test of group differences: $Q_b(1) = 1.51$, $p = 0.22$			
			0.001

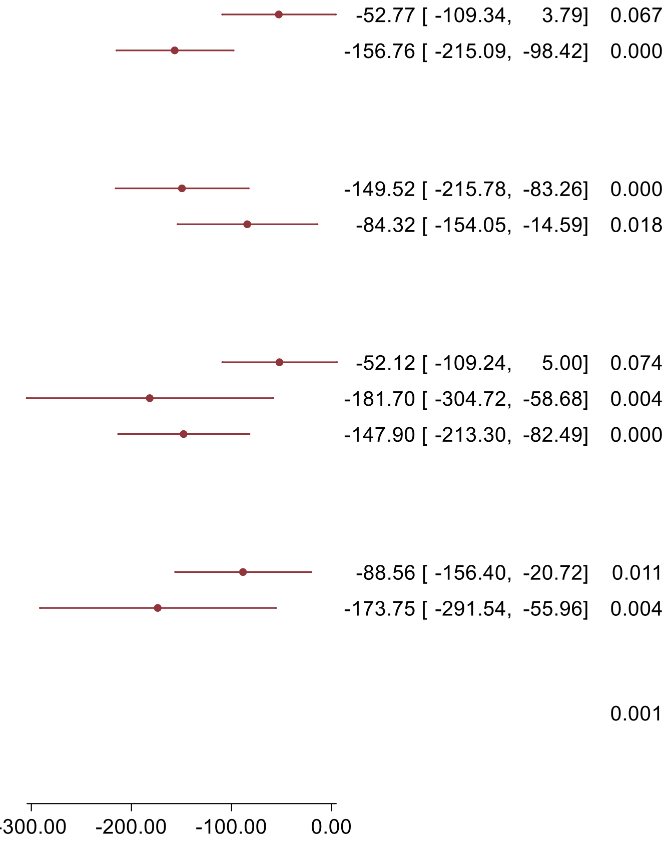

Random-effects REML model

NOTE: The risk of bias assessment was performed using the AMSTAR-2 tool, with ratings categorised as "high ", "moderate", "low", or "critically low" confidence.

Supplementary Figure 25. Subgroup analyses for weight (standardised mean difference).

Subgroup	No. of studies	Effect size with 95% CI	p-value
**Age**			
50 or less	4	-0.31 [-0.72, 0.09]	0.127
Not reported	1	-0.43 [-0.61, -0.25]	0.000
Over 50	2	-0.30 [-0.48, -0.12]	0.001
Test of group differences: $Q_b(2) = 1.07$, $p = 0.59$			
**Gender**			
Mixed	5	-0.30 [-0.51, -0.10]	0.004
Women	2	-0.44 [-1.31, 0.43]	0.323
Test of group differences: $Q_b(1) = 0.09$, $p = 0.77$			
**Population**			
Cancer	1	-0.35 [-0.48, -0.22]	0.000
Chronic diseases	1	-0.13 [-0.47, 0.21]	0.454
General population	2	-0.22 [-0.62, 0.18]	0.290
Overweight or obese	1	-0.57 [-0.75, -0.39]	0.000
Pregnancy	2	-0.44 [-1.31, 0.43]	0.323
Test of group differences: $Q_b(4) = 7.18$, $p = 0.13$			
**Intervention**			
Mixed	3	-0.17 [-0.39, 0.06]	0.141
Mobile apps only	1	-0.43 [-0.61, -0.25]	0.000
Mobile, SMS and web-based	2	-0.44 [-1.31, 0.43]	0.323
Web-based only	1	-0.57 [-0.75, -0.39]	0.000
Test of group differences: $Q_b(3) = 7.57$, $p = 0.06$			
**Risk of bias**			
Critically low	6	-0.34 [-0.56, -0.12]	0.003
Moderate	1	-0.13 [-0.47, 0.21]	0.454
Test of group differences: $Q_b(1) = 1.02$, $p = 0.31$			

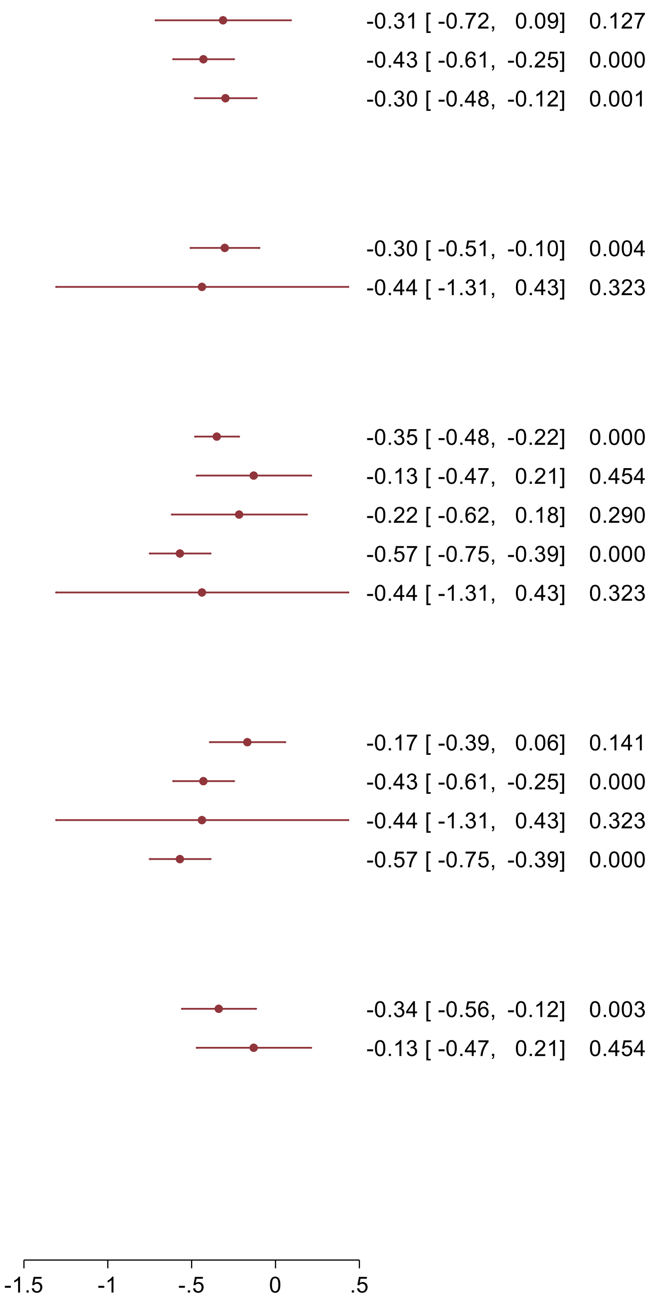

Random-effects REML model

NOTE: The risk of bias assessment was performed using the AMSTAR-2 tool, with ratings categorised as "high ", "moderate", "low", or "critically low" confidence.

Supplementary Figure 26. Subgroup analyses for weight (mean difference, kg)

Subgroup	No. of studies	Effect size with 95% CI	p-value
**Age**			
50 or less	7	-1.78 [-2.50, -1.05]	0.000
Not reported	2	-2.65 [-3.29, -2.02]	0.000
Over 50	2	-1.52 [-2.62, -0.42]	0.007
Test of group differences: $Q_b(2) = 4.72$, p = 0.09			
**Gender**			
Men	1	-2.27 [-3.67, -0.87]	0.001
Mixed	10	-1.87 [-2.43, -1.30]	0.000
Test of group differences: $Q_b(1) = 0.28$, p = 0.60			
**Population**			
Chronic disease	2	-2.13 [-2.68, -1.59]	0.000
General population	2	-1.50 [-2.67, -0.33]	0.012
Overweight or obese	7	-1.90 [-2.69, -1.10]	0.000
Test of group differences: $Q_b(2) = 1.00$, p = 0.61			
**Intervention**			
Mixed	3	-1.89 [-3.18, -0.60]	0.004
Mobile and web-based	2	-2.69 [-3.45, -1.93]	0.000
Mobile app, SMS and website	2	-1.52 [-2.62, -0.42]	0.007
Mobile apps only	3	-1.82 [-2.78, -0.86]	0.000
Web-based only	1	-0.56 [-4.59, 3.47]	0.785
Test of group differences: $Q_b(4) = 4.37$, p = 0.36			
**Risk of bias**			
Critically low	4	-1.46 [-2.41, -0.50]	0.003
High	3	-1.69 [-2.93, -0.46]	0.007
Low	4	-2.44 [-2.96, -1.92]	0.000
Test of group differences: $Q_b(2) = 3.73$, p = 0.16			

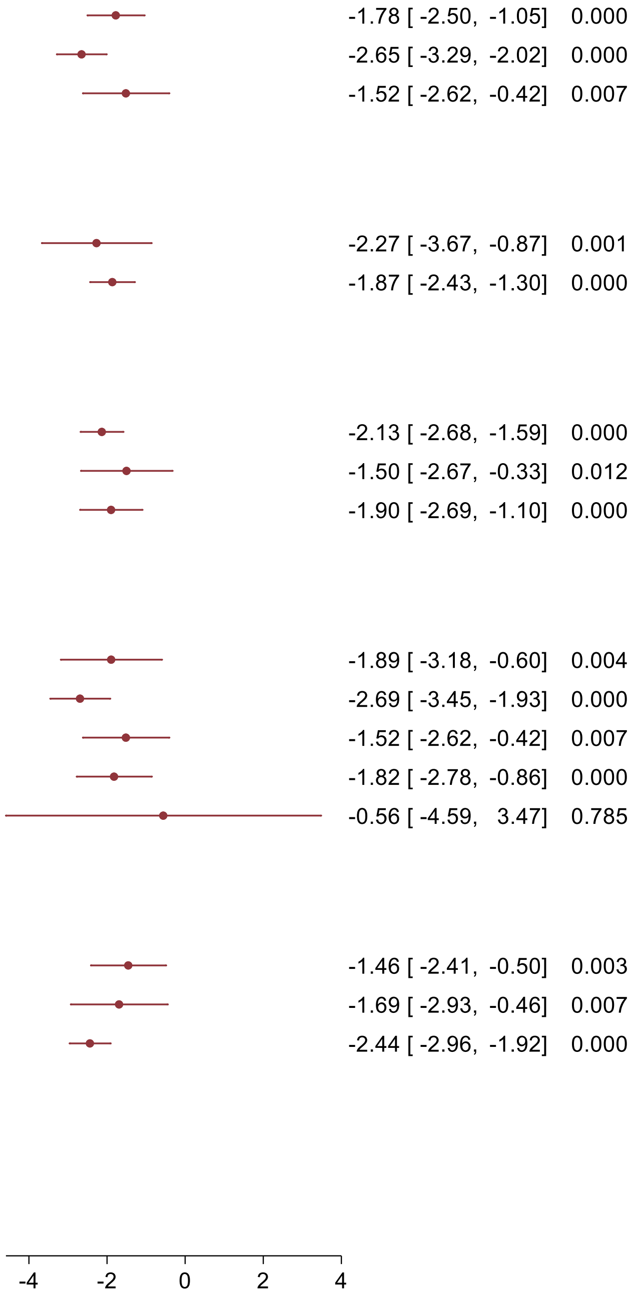

Random-effects REML model

NOTE: The risk of bias assessment was performed using the AMSTAR-2 tool, with ratings categorised as "high ", "moderate", "low", or "critically low" confidence.

Supplementary Table 3. Medline search strategy and terms

MEDLINE(R) ALL <1946 to November 12, 2021><br>Ovid MEDLINE(R) ALL <1946 to November 19, 2021>	
1 Meta-Analysis as Topic/<br>2 meta analy$.tw.<br>3 metaanaly$.tw.<br>4 Meta-Analysis/<br>5 Systematic Review/ [addition]<br>6 Systematic Reviews as Topic/ [addition]<br>7 (systematic adj (review$1 or overview$1)).tw.<br>8 exp Review Literature as Topic/<br>9 or/1-8<br>10 cochrane.ab.<br>11 embase.ab.<br>12 (psychlit or psyclit).ab.<br>13 (psychinfo or psycinfo).ab.<br>14 (cinahl or cinhal).ab.<br>15 science citation index.ab.<br>16 bids.ab.<br>17 cancerlit.ab.<br>18 or/10-17<br>19 reference list$.ab.<br>20 bibliograph$.ab.<br>21 hand-search$.ab.<br>22 relevant journals.ab.<br>23 manual search$.ab.<br>24 or/19-23<br>25 selection criteria.ab.<br>26 data extraction.ab.<br>27 25 or 26<br>28 Review/<br>29 27 and 28<br>30 Comment/<br>31 Letter/<br>32 Editorial/<br>33 animal/	34 human/<br>35 33 not (33 and 34) 36 or/30-32,35<br>37 9 or 18 or 24 or 29<br>38 37 not 36<br>39 exp exercise/<br>40 exp exercise therapy/<br>41 exp sports/<br>42 Physical Fitness/<br>43 (physical* adj5 (fit* or train* or activ* or endur* or exer*)).ti,ab.<br>44 (exercis* adj5 (train* or physical* or activ*)).ti,ab.<br>45 sport*.ti,ab.<br>46 walk*.ti,ab.<br>47 swim*.ti,ab.<br>48 pilates.ti,ab.<br>49 step*.ti,ab.<br>50 HIIT.ti,ab.<br>51 (tai ji or tai chi or tai-ji or tai-chi).ti,ab.<br>52 (resistance adj3 train*).ti,ab.<br>53 39 or 40 or 41 or 42 or 43 or 44 or 45 or 46 or 47 or 48 or 49 or 50 or 51 or 52<br>54 38 and 53<br>55 depress*.ti,ab.<br>56 anxiety.ti,ab.<br>57 distress.ti,ab.<br>58 55 or 56 or 57<br>59 54 and 58<br>60 food.ti,ab.<br>61 diet*.ti,ab.<br>62 nutriti*.ti,ab.<br>63 59 not (60 or 61 or 62)
